# Information nudging, attitude, and protective behaviours against Nipah virus in India, 2026: a national experimental survey

**DOI:** 10.7189/jogh.16.04362

**Published:** 2026-11-06

**Authors:** Sijia Wang, Liyan Zhou, Kunyue Xing, Zhiyu Zhang, Shunzhe Wu, Qiao Liu, Min Liu, Hongliang Zhang, Sri Harsha Kota, Liyuan Tao, Jue Liu

**Affiliations:** 1 Department of Epidemiology and Biostatistics, School of Public Health, Peking University, Beijing, China; 2 Health Emergency Management Centre, Peking University, Beijing, China; 3 Key Laboratory of Epidemiology of Major Diseases, Peking University, Ministry of Education, Beijing, China; 4 School of Environment and Architecture, University of Shanghai for Science and Technology, Shanghai, China; 5 Department of Civil Engineering, Indian Institute of Technology Delhi, New Delhi, India; 6 Research Centre of Clinical Epidemiology, Peking University Third Hospital, Beijing, China; 7 Institute for Global Health and Development, Peking University, Beijing, China

**Keywords:** Nipah virus, information nudge, health belief model, protective behaviour

## Abstract

**Background:**

Nipah virus (NiV) poses a significant public health threat due to its high fatality, yet public readiness for medical countermeasures remains understudied. We aimed to evaluate the associations of perceived severity and benefits information nudges with the dimensions of the Health Belief Model (HBM), identify highly responsive populations, and assess whether changes in HBM dimensions mediate the associations between information nudges and protective behavioural intentions in India.

**Methods:**

We conducted a nationwide experimental web survey among Indian adults between 6 and 18 February 2026. The survey contained an embedded information nudge module in which participants were randomly assigned to one of three groups: a perceived severity nudge, a perceived benefits nudge, or no nudge. We assessed changes in HBM dimensions using analysis of covariance. Six predictive models were evaluated to identify subpopulations highly susceptible to cognitive shifts. Mediation analyses were performed to estimate the indirect effects of nudges on intentions to promptly seek medical care, receive vaccination, and accept treatment.

**Results:**

Our sample comprised 1,124 participants randomly assigned to receive a perceived severity nudge (n = 373), a perceived benefits nudge (n = 376), or no nudge (n = 375). Compared with no nudge, the severity nudge significantly increased perceived severity (adjusted mean difference (AMD) = 0.17; 95% confidence interval (CI) = 0.05, 0.30; *P* = 0.005) and cues to action (AMD = 0.20; 95% CI = 0.09, 0.32; *P* < 0.001), while the benefits nudge significantly increased cues to action (0.30; 95% CI = 0.19, 0.41; *P* < 0.001). Predictive modelling (extreme gradient boosting: area under the receiver operating characteristic curve (AUROC) = 0.717; logistic regression: AUROC = 0.789) identified baseline health beliefs and residence outside prior outbreak regions as dominant contributors to nudge responsiveness. Mediation analyses indicated significant indirect effects of the severity nudge on treatment intention *via* perceived severity (effect estimate (*β*) = 0.016; 95% CI = 0.002, 0.032), as well as the perceived benefits nudge *via* cues to action on both vaccination intention (*β* = 0.024; 95% CI = 0.001, 0.054) and treatment intention (*β*  = 0.028; 95% CI = 0.002, 0.055). Neither information nudge significantly influenced promptly seeking medical care.

**Conclusions:**

Targeted information nudges could alter health beliefs and influence vaccination and treatment readiness through distinct psychological pathways.

Nipah virus (NiV) is a highly pathogenic zoonotic paramyxovirus of the genus *Henipavirus* for which fruit bats of the genus *Pteropus* serve as the natural reservoirs [1]. It typically infects humans by way of direct contact with infected animals or the consumption of bat-contaminated food products such as raw date palm juice, and can subsequently propagate *via* sustained human-to-human transmission, particularly in healthcare and household settings [2]. Since its initial identification during the 1998–1999 outbreak in Malaysia, NiV has caused recurrent outbreaks across South and Southeast Asia, including Malaysia, Singapore, Bangladesh, India, and the Philippines [3,4]. A re-emergence was identified in early 2026 in West Bengal, India [5], and Bangladesh [6], indicating continued sporadic transmission in previously affected regions.

NiV infection presents with a broad range of symptoms and complications, from subclinical or mild febrile illness to severe respiratory disease and acute encephalitis [7]. The reported case fatality ratios are alarmingly high, ranging from approximately 40% to 75%, partly reflecting differences in case ascertainment and access to timely supportive care [8]. Beyond acute mortality, long-term neurological sequelae have been documented among survivors, including relapse encephalitis and persistent cognitive dysfunction, contributing to an under-recognised burden of morbidity [9,10].

Despite its high lethality and outbreak potential, there are currently no licensed vaccines or specific therapeutics available for NiV [11], which has been recognised by the World Health Organization (WHO) as a research priority [8]. Several candidate vaccines, including mRNA and viral vector platforms, as well as therapeutic agents such as monoclonal antibodies and small molecules, have demonstrated promising immunogenicity and efficacy in preclinical models [11,12], while a phase II trial of the ChAdOx1 NipahB vaccine has recently been initiated in Bangladesh [13].

Simultaneously, low public knowledge regarding NiV transmission and prevention in regions with prior NiV outbreaks remains a critical challenge. Fewer than one-third of participants in recent surveys demonstrated adequate understanding of NiV [14]. Raw date palm sap consumption remains common in affected communities [14], while knowledge gaps regarding this transmission route persist even among healthcare providers [15]. Intention to promptly seek medical care likewise remains low, with only about a third of individuals reporting they would seek medical advice upon experiencing symptoms [14]. Low knowledge and misleading perceptions have also been associated with suboptimal attitudes towards vaccines and preventive behaviours in other emerging infectious diseases [16], and both risk perception and perceived benefits have been shown to strongly influence vaccination acceptance during epidemics [17–19]. Given that NiV vaccines and therapeutics are under development, proactively assessing and improving population-level readiness for and acceptance of such interventions emerges as a public health imperative.

Targeted public health communication grounded in established behavioural theories could be crucial for advancing such behaviour. The Health Belief Model (HBM), one of the most widely used frameworks in health behaviour research, posits that individuals’ engagement in health-related actions is shaped by their perceived susceptibility to and severity of a disease, perceived benefits and barriers to action, cues to action, and self-efficacy [20]. In the context of emerging infectious diseases such as COVID-19, research has identified HBM constructs as significant predictors of vaccination uptake and other preventive behaviours [21,22]. However, few studies have applied HBM specifically to NiV, and few have used predictive modelling to identify subpopulations highly responsive to such cognitive interventions. Furthermore, little is known about how targeted information nudges, specifically the perceived severity and the perceived benefits nudges, influence NiV-related health beliefs and subsequent readiness to accept future medical countermeasures.

To generate evidence for the development of effective public health messaging, we aimed to evaluate the associations of perceived severity and perceived benefits nudges with HBM dimensions, use predictive modelling to identify populations highly responsive to these nudges, and assess whether changes in HBM dimensions mediate the associations between information nudges and intentions to promptly seek medical care, receive a vaccine, and receive treatment.

## METHODS

We conducted a nationwide, experimental web-based survey with an embedded information nudge between 6 and 18 February 2026 among adults aged 18 years and older across all 36 states and union territories in India. We recruited participants through Dynata, a professional international commercial web panel platform [23]. Keeping in mind the lack of robust historical data on public readiness to accept future NiV vaccines and therapeutics, as well as baseline rates for promptly seeking medical care, we calculated the sample size with a conservative expected rate of 0.50 for all three primary outcomes and an allowable error of 0.07 times the expected rate, setting the two-sided alpha at 0.05, resulting in a calculated minimum target sample size of 810 respondents. To account for potential invalid responses, we expanded the base sample size to an initial recruitment target of 1000 respondents, whom we then allocated using a proportional quota sampling strategy across the geographic regions, in accordance with projected population data derived from the 2011 Indian National Census [24], *i.e. *the most recent complete official census available. To ensure sufficient statistical power and a balanced allocation across the three information nudge groups, we adjusted the minimum quota for any individual state to at least three respondents. We set the quotas for Kerala and West Bengal at 100 respondents each due to their epidemiological significance and history of NiV outbreaks [5,25]. The final required sample size was determined to be 1113 respondents. We performed these calculations in PASS 2025, version 25.0.5 (NCSS LLC., Kaysville, Utah, USA).

Afterwards, Dynata randomly distributed email invitations according to the sampling frame and implemented integrated quality-control measures, including restriction to unique Indian IP addresses and automated screening that would identify excessively short completion times or straight-lining patterns. Only participants who provided informed consent could continue to the survey.

### Survey instrument

We developed an anonymised self-administered questionnaire in English and Hindi (**Online Supplementary Document**) that first gathered the following sociodemographic data: age, gender, state of residence, residence type (rural or urban), educational level, occupation, and the presence of vulnerable family members (*e.g.* children, older adults, pregnant women). The questionnaire then captured NiV-specific epidemiological and behavioural risks (participants’ frequency of handling raw pork, frequency of drinking unboiled raw date palm sap, and average number of close contacts per day) and prior cognitive awareness and exposure data (prior awareness of the NiV, exposure to NiV-related information in the past 12 months, and most trusted sources of health information (*e.g.* health departments, healthcare institutions, media, or social networks)).

We developed six items to assess objective NiV-related knowledge based on current World Health Organization fact sheets and guidelines [2]. These items covered bat-related exposure, including contaminated food; person-to-person transmission through close contact or caregiving; fever and respiratory symptoms; severe neurological symptoms; the availability of specific curative medicines; and the benefits of promptly seeking medical care when severe symptoms occur. Participants were asked to judge each statement as true, false, or not sure. For example, one item stated, ‘There are currently widely available specific medicines that can “cure” Nipah virus infection in most cases’ (correct answer: false). To quantify objective knowledge, each correct answer was awarded one point, with one item being reverse scored, resulting in a total score ranging from 0 to 6, where higher scores indicate better knowledge. The scale yielded a Kuder–Richardson 20 (KR-20) coefficient of 0.577. However, given that the items assessed distinct factual domains of NiV knowledge, rather than interchangeable manifestations of a single construct, high inter-item correlations were not necessarily expected [26–28].

We employed a separate six-item scale grounded in the HBM to evaluate the psychological determinants of protective behaviours. Both at baseline before the nudge and immediately after the information exposure, participants evaluated statements on a five-point Likert scale ranging from 1 (strongly disagree) to 5 (strongly agree) across six core constructs [29,30]:

− perceived susceptibility: an individual’s belief regarding their personal risk of NiV infection;

− perceived severity: beliefs about the seriousness of its consequences, such as hospitalisation or death;

− perceived benefits: beliefs in the effectiveness of recommended protective measures in reducing infection risk;

− perceived barriers: perceived psychological costs, specifically worries about facing social stigma or negative comments when seeking care;

− cues to action: external triggers that activate readiness to act, encompassing the receipt of clear preventive guidance from the media, community campaigns, or healthcare workers;

− self-efficacy: confidence in ability to successfully execute the advised protective behaviours.

In the information nudge module, participants were randomly assigned to one of three groups (in a 1:1:1 ratio), with the sampling process stratified by state of residence using pre-established quotas. Group A (perceived severity nudge) received a message emphasising the severe threat of NiV, detailing its high case-fatality rate (40–75%) and severe clinical consequences, such as seizures, encephalitis, and coma. Group B (perceived benefits nudge) received a message highlighting the benefits of actionable preventive behaviours, such as avoiding unboiled date palm sap, washing fruit, and wearing masks, in reducing infection risk and protecting family members. Group C (no nudge) received no NiV-related information and instead took a 30-second break featuring a neutral landscape image.

The primary behavioural outcomes were a binary measure of intention to seek medical care promptly if symptoms occur, vaccination intention, and treatment intention. We assessed the first outcome based on whether participants selected the corresponding option in a multiple-response question about protective measures following a hypothetical local NiV case announcement. We assessed vaccination and treatment intentions using two separate single-choice questions asking whether participants would be willing to receive a safe and effective NiV vaccine or treatment if available in the future. The latter two behavioural intentions were assessed using five ordinal response options ranging from ‘very willing’ to ‘very unwilling’.

We defined two secondary analytical outcomes representing positive cognitive shifts for predictive modelling, operationalising them as binary variables indicating any post-nudge increase (*i.e.* a score change >0) in perceived severity and perceived benefits. Given that we assessed all HBM items using a single five-point Likert item, score changes occur in discrete steps between response categories (1 = strongly disagree, 2 = disagree, 3 = not sure, 4 = agree, 5 = strongly agree), and any increase represents a shift to a higher response level.

### Data analysis

We described our sample’s baseline characteristics (**Online Supplementary Document**) using means and standard deviations (SDs) for continuous variables and frequencies and percentages for categorical variables. To evaluate the associations of the information nudges with the HBM dimensions, we employed analysis of covariance to compare post-nudge scores across groups, adjusting for the corresponding baseline scores. We calculated global F-tests, followed by pairwise comparisons (perceived severity nudge *vs* no nudge; perceived benefits nudge *vs* no nudge) using estimated marginal means with Bonferroni adjustments.

We then developed predictive models to identify subpopulations highly susceptible to the desired cognitive changes (defined as an increase in perceived severity or perceived benefits), employing six algorithms: extreme gradient boosting (XGBoost), artificial neural network (ANN), random forest, least absolute shrinkage and selection operator, support vector machine, and logistic regression. These models incorporated 11 baseline behavioural and demographic variables: corresponding baseline HBM dimension score, frequency of handling raw pork, frequency of drinking raw date palm sap, daily close contacts, most trusted information source, objective knowledge score about NiV, residence in regions with prior NiV outbreaks, gender, age, education level, and presence of vulnerable family members. We evaluated model performance using the area under the receiver operating characteristic curve (AUROC) with 95% confidence intervals (CIs) through 10-fold cross-validation.

To explore whether and how each HBM dimension mediates the effect of the information nudges on final protective behaviours, we utilised the *mediation* package to conduct mediation analyses. Specifically, we estimated the indirect effects of the information nudges on promptly seeking medical care, vaccination intention, and treatment intention through all six HBM dimensions. These mediation models were adjusted for the same 11 baseline behavioural and demographic variables utilised in the predictive modelling.

Lastly, we conducted sensitivity analyses for both the predictive and mediation models to test the robustness of our results. Specifically, to evaluate the predictive modelling algorithms’ real-world applicability without relying on extensive epidemiological surveys, we performed a sensitivity analysis using a minimal feature set consisting exclusively of the corresponding baseline HBM dimension score, residence in regions with prior NiV outbreaks, gender, age, education level, and the presence of vulnerable family members. We then conducted three sensitivity analyses for the mediation models. First, to rule out over-fitting or collider bias introduced by extensive covariates, we estimated a minimal covariate adjustment model controlling for the identical minimal feature set described above. Second, to mitigate potential measurement bias from the objective knowledge score, we substituted this single metric with two subjective prior information exposure variables (prior awareness of the NiV and information exposure in the past 12 months), while retaining all other covariates from the primary model. Finally, we replaced specific behavioural exposures (frequency of handling raw pork, drinking raw date palm sap, and daily close contacts) with generalised high-risk exposure histories (recent high-risk experiences in the past 3 months and high-risk venue exposures in the past 12 months) to confirm that different epidemiological categorisations did not alter the core mediation pathways, keeping the remaining primary covariates unchanged.

We conducted all analyses in *R*, version 4.4.1 (R Foundation for Statistical Computing, Vienna, Austria). A two-sided *P*-value <0.05 denoted statistical significance.

## RESULTS

Of the 2,804 individuals who accessed the survey, 1,124 completed the full questionnaire and met all inclusion criteria, yielding a completion rate of 40.1% (Figure S1 in the **Online Supplementary Document**). Participants were then randomly presented with one of three information nudges: 373 received the perceived severity nudge, 376 received the perceived benefits nudge, and 375 received no information nudge. Following minor exclusions due to sparse covariate categories, we retained 368, 371, and 373 participants for the three nudge groups, respectively, for the predictive modelling and mediation analyses.

Of the 1,124 participants, 51.3% were male, 74.9% resided in urban areas, 30.7% were over 40 years old, 88.7% had completed tertiary education or higher, 49.6% were company employees or service industry workers, and 82.0% lived with vulnerable family members. Regarding potential exposure risks, 30.6% reported handling raw pork and 34.5% reported drinking raw date palm sap at least monthly, while 22.0% had more than five close contacts per day. Most participants (66.4%) were familiar with the NiV, while 72.6% had been exposed to NiV-related information in the past 12 months. The overall mean baseline NiV knowledge score was 4.07 (SD = 1.46). The most trusted sources of information were health departments or government announcements (41.9%), followed by doctors and healthcare institutions (31.2%). Regarding protective behaviours, 70.9% of participants intended to seek medical care promptly if symptoms occur and report any exposure history, with high levels for vaccination intention (mean (x̄) = 4.30, SD = 0.79) and treatment intention (x̄ = 4.35, SD = 0.73). The three nudge groups were well balanced in terms of baseline demographic data, prior knowledge on NiV, and pre-nudge health beliefs (**Table 1**).

**Table 1:** Baseline characteristics, knowledge, pre-nudge health beliefs, and protective behavioural outcomes of the study population by information nudge groups (n = 1124)

	Total (n = 1124, 100.0%)	Perceived severity nudge (n = 373, 33.2%)	Perceived benefits nudge (n = 376, 33.5%)	No nudge (n = 375, 33.4%)
**Age in years**				
≤25	311 (27.7)	119 (31.9)	97 (25.8)	95 (25.3)
26–30	169 (15.0)	57 (15.3)	63 (16.8)	49 (13.1)
31–35	159 (14.1)	44 (11.8)	61 (16.2)	54 (14.4)
36–40	140 (12.5)	40 (10.7)	36 (9.6)	64 (17.1)
>40	345 (30.7)	113 (30.3)	119 (31.6)	113 (30.1)
**Gende**r				
Male	577 (51.3)	198 (53.1)	184 (48.9)	195 (52.0)
Female	546 (48.6)	175 (46.9)	192 (51.1)	179 (47.7)
Third gender/transgender/prefer not to disclose	1 (0.1)	0 (0.0)	0 (0.0)	1 (0.3)
**Residence type**				
Rural	282 (25.1)	87 (23.3)	96 (25.5)	99 (26.4)
Urban	842 (74.9)	286 (76.7)	280 (74.5)	276 (73.6)
**Highest education**				
Primary school or below	5 (0.4)	2 (0.5)	0 (0.0)	3 (0.8)
Secondary school	122 (10.9)	42 (11.3)	33 (8.8)	47 (12.5)
Junior college/bachelor’s	565 (50.3)	191 (51.2)	199 (52.9)	175 (46.7)
Master’s degree or above	432 (38.4)	138 (37.0)	144 (38.3)	150 (40.0)
Occupation				
Healthcare worker	61 (5.4)	23 (6.2)	15 (4.0)	23 (6.1)
Veterinarian/animal-related professional	6 (0.5)	1 (0.3)	1 (0.3)	4 (1.1)
Livestock farming/breeding/slaughtering/meat processing-related	16 (1.4)	4 (1.1)	6 (1.6)	6 (1.6)
Student	169 (15.0)	68 (18.2)	59 (15.7)	42 (11.2)
Company employee/service industry worker	558 (49.6)	178 (47.7)	186 (49.5)	194 (51.7)
Unemployed/homemaker/caregiver	110 (9.8)	32 (8.6)	33 (8.8)	45 (12.0)
Other	204 (18.1)	67 (18.0)	76 (20.2)	61 (16.3)
Vulnerable family members				
Yes (children/elderly/pregnant/sick)	922 (82.0)	307 (82.3)	316 (84.0)	299 (79.7)
No	202 (18.0)	66 (17.7)	60 (16.0)	76 (20.3)
Average close contacts per day				
≤5 times/d	782 (69.6)	268 (71.8)	261 (69.4)	253 (67.5)
>5 times/d	247 (22.0)	74 (19.8)	84 (22.3)	89 (23.7)
Not sure	95 (8.5)	31 (8.3)	31 (8.2)	33 (8.8)
Handling raw pork				
Yes (≥1–3 times/mo)	344 (30.6)	115 (30.8)	119 (31.6)	110 (29.3)
Rarely/never	780 (69.4)	258 (69.2)	257 (68.4)	265 (70.7)
Drinking raw date palm sap				
Yes (≥1–3 times/mo)	388 (34.5)	126 (33.8)	124 (33.0)	138 (36.8)
Rarely/never	736 (65.5)	247 (66.2)	252 (67.0)	237 (63.2)
Heard of NiV				
Never/don’t know much	378 (33.6)	130 (34.9)	120 (31.9)	128 (34.1)
Know a little/fairly well	746 (66.4)	243 (65.1)	256 (68.1)	247 (65.9)
NiV information exposure in past 12 months				
No/not sure	308 (27.4)	102 (27.3)	96 (25.5)	110 (29.3)
Yes (once/multiple times)	816 (72.6)	271 (72.7)	280 (74.5)	265 (70.7)
**Most trusted information source**				
Health department/government announcements	471 (41.9)	149 (39.9)	155 (41.2)	167 (44.5)
Doctors/healthcare institutions	351 (31.2)	126 (33.8)	114 (30.3)	111 (29.6)
Television/radio/newspapers	130 (11.6)	44 (11.8)	42 (11.2)	44 (11.7)
Social media	90 (8.0)	29 (7.8)	33 (8.8)	28 (7.5)
Family/friends/community	71 (6.3)	20 (5.4)	27 (7.2)	24 (6.4)
Other/not sure	11 (1.0)	5 (1.3)	5 (1.3)	1 (0.3)
Baseline NiV knowledge score (range = 0–6), x̄ (SD)	4.07 (1.46)	4.08 (1.39)	4.01 (1.48)	4.11 (1.50)
**Pre-nudge health beliefs (1–5 Likert scale), x̄ (SD)**				
Perceived susceptibility	2.60 (1.26)	2.61 (1.28)	2.62 (1.24)	2.57 (1.25)
Perceived severity	3.57 (1.10)	3.58 (1.12)	3.62 (1.08)	3.51 (1.10)
Perceived benefits	4.17 (0.91)	4.17 (0.95)	4.20 (0.86)	4.16 (0.94)
Perceived barriers	2.94 (1.19)	2.94 (1.23)	2.90 (1.19)	2.99 (1.16)
Self-efficacy	4.18 (0.85)	4.21 (0.82)	4.19 (0.80)	4.13 (0.92)
Cues to action	3.55 (1.05)	3.54 (1.06)	3.60 (1.04)	3.50 (1.06)
**Protective behaviour**				
Promptly seeking medical care				
*Yes*	797 (70.9)	266 (71.3)	266 (70.7)	265 (70.7)
*No*	327 (29.1)	107 (28.7)	110 (29.3)	110 (29.3)
Vaccination intention (range = 1–5), x̄ (SD)	4.30 (0.79)	4.28 (0.84)	4.34 (0.76)	4.27 (0.78)
Treatment intention (range = 1–5), x̄ (SD)	4.35 (0.73)	4.38 (0.74)	4.36 (0.74)	4.33 (0.71)

NiV – Nipah virus, SD – standard deviation, x̄ – mean

*Presented as n (%) unless specified otherwise.

Compared with no nudge, the perceived severity nudge significantly increased perceived severity (adjusted mean difference (AMD) = 0.17; 95% CI = 0.05, 0.30; *P* = 0.005) and cues to action (AMD = 0.20; 95% CI = 0.09, 0.32; *P* < 0.001). The perceived benefits nudge yielded a significant increase in cues to action (AMD = 0.30; 95% CI = 0.19–0.41; *P* < 0.001) relative to the no nudge group, but did not significantly alter perceived severity (AMD = 0.11; 95% CI = −0.01, 0.24; *P* = 0.094). We observed no significant pairwise differences between either nudge and the no nudge group regarding perceived susceptibility, perceived benefits, perceived barriers, or self-efficacy following Bonferroni adjustment (**Table 2**).

**Table 2:** Estimated effects of information nudges on HBM dimensions (range = 1–5)*

	Baseline, x̄ (SD)	Post-nudge, x̄ (SD)	AMD *vs*. no nudge (95% CI)	*P*-value
**Total HBM score (range = 6–30)**				<0.001
Perceived severity nudge	21.17 (3.15)	21.68 (3.31)	0.64 (0.31, 0.97)	<0.001
Perceived benefits nudge	21.33 (2.95)	21.82 (2.88)	0.66 (0.33, 0.98)	<0.001
No nudge	20.88 (3.04)	20.81 (3.02)	ref	
**Perceived susceptibility**				0.227
Perceived severity nudge	2.61 (1.28)	2.64 (1.31)	0.05 (−0.08, 0.18)	0.773
Perceived benefits nudge	2.62 (1.24)	2.65 (1.24)	0.06 (−0.08, 0.19)	0.683
No nudge	2.57 (1.25)	2.56 (1.25)	ref	
**Perceived severity**				<0.001
Perceived severity nudge	3.58 (1.12)	3.72 (1.10)	0.17 (0.05, 0.30)	0.005
Perceived benefits nudge	3.62 (1.08)	3.68 (1.10)	0.11 (−0.01, 0.24)	0.094
No nudge	3.51 (1.10)	3.49 (1.09)	ref	
**Perceived benefits**				0.069
Perceived severity nudge	4.17 (0.95)	4.25 (0.84)	0.09 (−0.01, 0.20)	0.105
Perceived benefits nudge	4.20 (0.86)	4.25 (0.83)	0.07 (−0.04, 0.17)	0.279
No nudge	4.16 (0.94)	4.15 (0.94)	ref	
**Perceived barriers**				0.049
Perceived severity nudge	2.94 (1.23)	2.91 (1.24)	−0.06 (−0.19, 0.07)	0.658
Perceived benefits nudge	2.90 (1.19)	2.88 (1.23)	−0.06 (−0.19, 0.07)	0.535
No nudge	2.99 (1.16)	3.01 (1.16)	ref	
**Cues to action**				<0.001
Perceived severity nudge	3.54 (1.06)	3.72 (1.00)	0.20 (0.09, 0.32)	<0.001
Perceived benefits nudge	3.60 (1.04)	3.86 (0.99)	0.30 (0.19, 0.41)	<0.001
No nudge	3.50 (1.06)	3.49 (1.07)	ref	
**Self-efficacy**				0.003
Perceived severity nudge	4.21 (0.82)	4.27 (0.81)	0.08 (−0.02, 0.18)	0.137
Perceived benefits nudge	4.19 (0.80)	4.25 (0.77)	0.08 (−0.02, 0.18)	0.147
No nudge	4.13 (0.92)	4.13 (0.92)	ref	

AMD – adjusted mean difference, CI – confidence interval, HBM – Health Belief Model, ref – reference SD – standard deviation, x̄ – mean

*The perceived severity nudge, perceived benefits nudge, and no nudge groups comprised 373, 376, and 375 participants, respectively.

For predicting increased perceived severity, the AUROCs for the artificial neural network (Figure S2A and Table S1 in the **Online Supplementary Document**), least absolute shrinkage and selection operator, logistic regression, random forest, support vector machine, and XGBoost models were 0.709, 0.689, 0.710, 0.697, 0.637, and 0.717, respectively, meaning that the XGBoost model demonstrated the highest discriminative ability (AUROC = 0.717; 95% CI = 0.654, 0.779). For predicting increased perceived benefits (Figure S2B and Table S1 in the **Online Supplementary Document**), the AUROCs for the six models were 0.767, 0.768, 0.789, 0.736, 0.784, and 0.776, with logistic regression achieving the optimal performance (AUROC = 0.789; 95% CI = 0.727, 0.850). Consequently, we selected the XGBoost and logistic regression models to extract feature importance and identify the baseline predictors driving nudge responsiveness (**Figure 1**).

**Figure 1:**
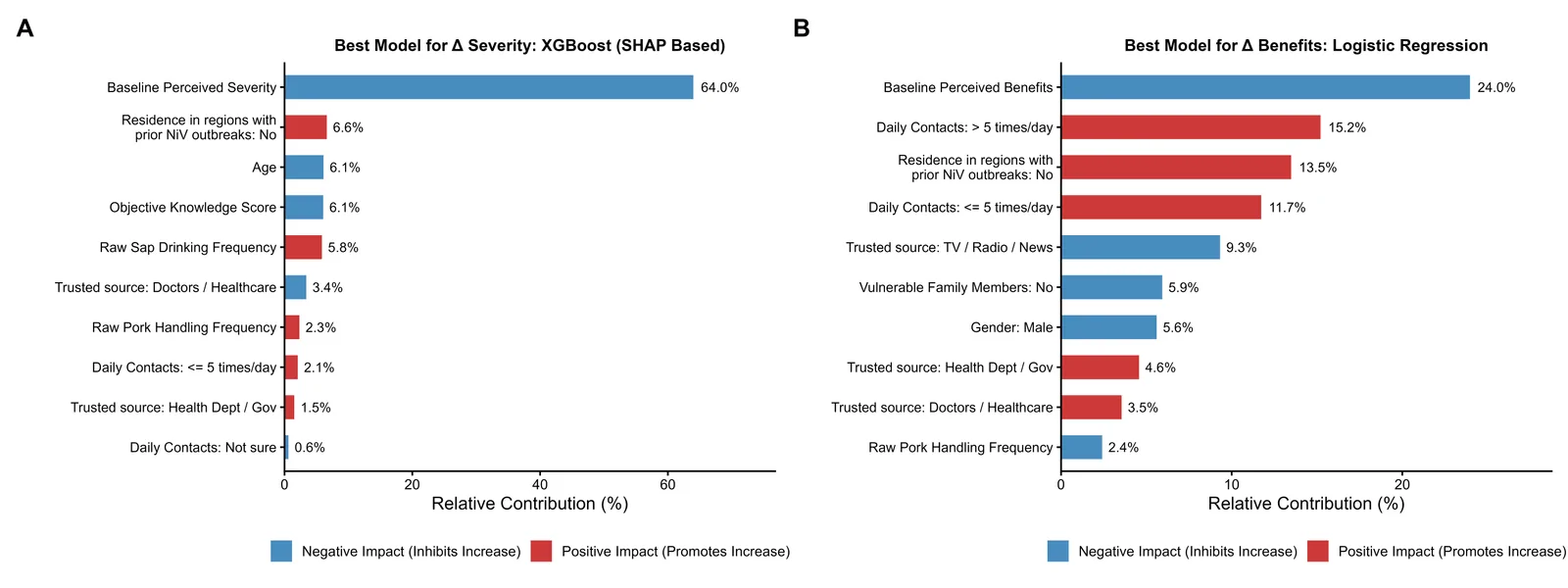
Directional feature importance and relative contributions of the best-performing models for predicting increased perceived severity and benefits. Models were adjusted for the corresponding baseline HBM dimension score (perceived severity or perceived benefits), frequency of handling raw pork, frequency of drinking raw date palm sap, daily close contacts, most trusted information source, objective knowledge score about NiV, residence in regions with prior NiV outbreaks, gender, age, education level, and presence of vulnerable family members. **Panel A.** XGBoost model for predicting increased perceived severity. Relative contribution is derived from mean absolute SHAP values, with directional impact determined by the Spearman correlation between feature values and their corresponding SHAP values. **Panel B.** Logistic regression model for predicting increased perceived benefits. Relative contribution represents the proportion of each absolute coefficient relative to the sum of all absolute coefficients, with directional impact indicated by the coefficient’s sign. HBM – Health Belief Model, NiV – Nipah virus, SHAP – Shapley additive explanations, XGBoost – extreme gradient boosting.

Feature importance analysis of the optimal models identified baseline HBM dimension scores as the dominant negative predictors of nudge responsiveness (**Figure 1**). In the XGBoost model for increased perceived severity, baseline perceived severity yielded the highest relative contribution (64.0%, negative impact), followed by not residing in regions with prior NiV outbreaks (6.6%, positive impact), age (6.1%, negative impact), and objective knowledge score (6.1%, negative impact). Similarly, the logistic regression model identified baseline perceived benefits as the strongest predictor for increased perceived benefits (24.0%, negative impact), followed by having more than five daily close contacts (15.2%, positive impact) and not residing in regions with prior NiV outbreaks (13.5%, positive impact).

Mediation analyses showed that, after adjusting for covariates, the total effects of the information nudges on promptly seeking medical care, vaccination intention, and treatment intention were not statistically significant. The perceived severity nudge yielded a significant indirect effect on treatment intention specifically *via* perceived severity (effect estimate (*β*) = 0.016; 95% CI = 0.002, 0.032); it significantly increased perceived severity (path a, representing the effect of the information nudge on the mediator: *β* = 0.170; 95% CI = 0.071, 0.269), while the direct effect remained non-significant (*β* = 0.043; 95% CI = −0.051, 0.141). Its indirect effects *via* perceived severity on promptly seeking medical care and vaccination intention were not statistically significant. Conversely, for the perceived benefits nudge, the indirect pathways *via* perceived benefits were not statistically significant across all three outcomes.

Further analyses identified distinct significant indirect pathways driven by cues to action. The perceived benefits nudge produced significant indirect effects *via* cues to action on both vaccination intention (indirect effect: *β* = 0.024; 95% CI = 0.001, 0.054; path a: *β* = 0.289; 95% CI = 0.204, 0.375; direct effect: *β* = 0.042; 95% CI = −0.063, 0.145) and treatment intention (indirect effect: *β* = 0.028; 95% CI = 0.002, 0.055; path a: *β* = 0.289; 95% CI = 0.204, 0.375; direct effect: *β* = 0.016; 95% CI = −0.077, 0.118). All other estimated indirect pathways through the remaining HBM dimensions across the three outcomes were not statistically significant (**Figure 2**, **Figure 3**; Figure S3 and Table S2 in the **Online Supplementary Document**).

**Figure 2:**
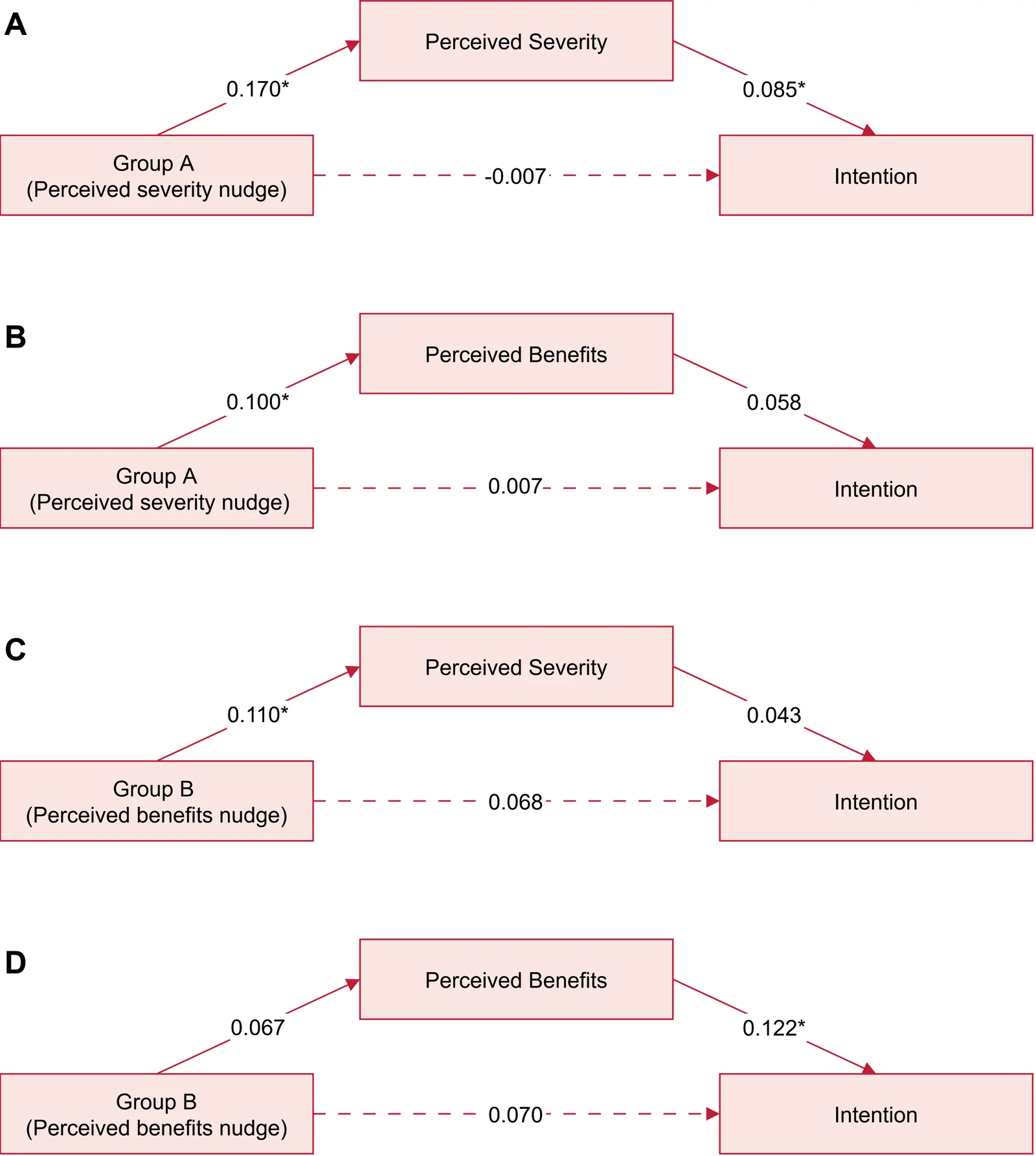
Mediation pathways of information nudges on vaccination intention. Values on the arrows represent path coefficients (*β*). Solid arrows indicate the indirect pathways mapping the effect of the information nudge on the mediator (path a) and the mediator on the outcome (path b). The dashed arrow indicates the direct effect of the information nudge on the outcome. Models were adjusted for the corresponding baseline HBM dimension score (perceived severity or perceived benefits), frequency of handling raw pork, frequency of drinking raw date palm sap, daily close contacts, most trusted information source, objective knowledge score about NiV, residence in regions with prior NiV outbreaks, gender, age, education level, and presence of vulnerable family members. HBM – Health Belief Model, NiV – Nipah virus. *P < 0.05.

**Figure 3:**
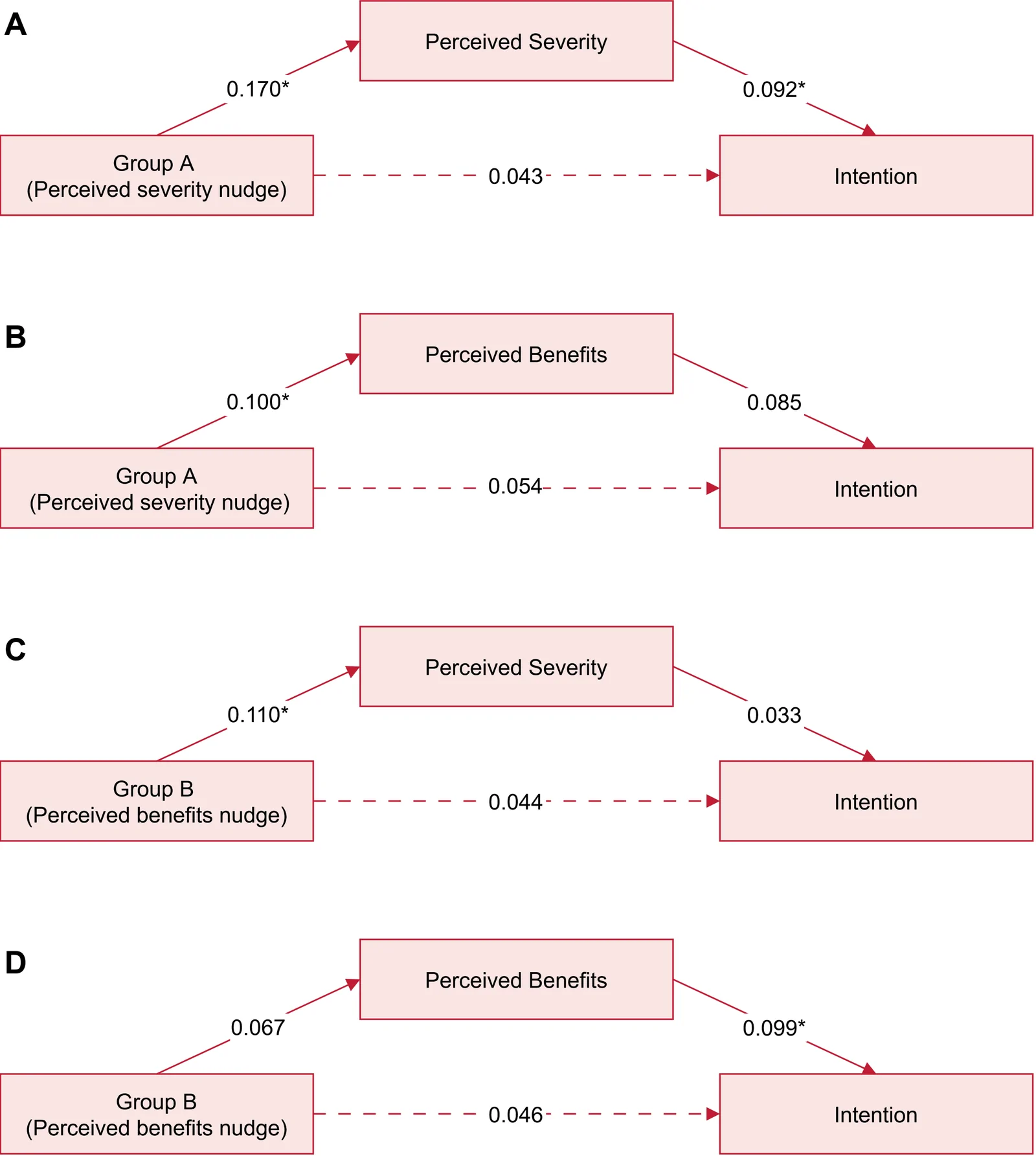
Mediation pathways of information nudges on treatment intention. Values on the arrows represent path coefficients (*β*). Solid arrows indicate the indirect pathways mapping the effect of the information nudge on the mediator (path a) and the mediator on the outcome (path b). The dashed arrow indicates the direct effect of the information nudge on the outcome. Models were adjusted for the corresponding baseline HBM dimension score (perceived severity or perceived benefits), frequency of handling raw pork, frequency of drinking raw date palm sap, daily close contacts, most trusted information source, objective knowledge score about NiV, residence in regions with prior NiV outbreaks, gender, age, education level, and presence of vulnerable family members. HBM – Health Belief Model, NiV – Nipah virus. *P < 0.05.

In sensitivity analyses (Tables S3–6 in the **Online Supplementary Document**), the predictive performances of the models remained stable or slightly improved when restricted to a minimal feature set. Logistic regression and artificial neural network achieved the highest AUROCs of 0.760 (95% CI = 0.699, 0.822) and 0.832 (95% CI = 0.778, 0.885) for predicting increased perceived severity and perceived benefits, respectively. The estimated indirect pathways remained robust for the mediation analyses, as well, with results being largely stable under the minimal covariate adjustment set, with only marginal shifts in 95% CIs. Furthermore, the significant indirect effects of the perceived severity nudge on treatment intention *via* perceived severity and the perceived benefits nudge on vaccination and treatment intentions *via* cues to action were consistently maintained when substituting the objective knowledge score with subjective cognitive proxies and when applying generalised high-risk exposure histories.

## DISCUSSION

This study offers insights into population-level health beliefs and protective behavioural intentions regarding the NiV, as well as their psychological mechanisms following targeted information nudges in India. Our findings indicate that the perceived severity nudge increased both perceived severity and cues to action, while the perceived benefits nudge significantly enhanced only cues to action. Predictive modelling identified baseline health belief scores and residence outside regions with prior NiV outbreaks as the dominant shared predictors of nudge responsiveness, alongside specific factors such as age and objective knowledge score for increased perceived severity, and high daily close contact frequency for increased perceived benefits. Mediation analyses showed that the perceived severity nudge indirectly promoted treatment intention specifically by elevating perceived severity, while the perceived benefits nudge indirectly drove both vaccination and treatment intentions by functioning as a significant cue to action.

Our analyses elucidate the specific cognitive shifts induced by the information nudges within the HBM framework. The perceived severity nudge successfully increased perceived severity, aligning with previous evidence indicating that threat-based communications are particularly effective at elevating risk appraisal for high case fatality rate diseases such as Ebola and MERS [31,32]. In our analysis, both the perceived severity and perceived benefits nudges significantly amplified cues to action, suggesting that the provision of health information itself inherently increases cues to action, regardless of information nudge, a finding consistent with prior studies showing that informational and educational interventions can significantly elevate cues to action by increasing exposure to health-related messages and reminders [33]. Conversely, the perceived benefits nudge did not significantly enhance perceived benefits, potentially due to the exceptionally high pre-nudge baseline scores for this dimension in our sample (x̄ = 4.17 out of 5). This ceiling effect may also be accompanied by a tendency for specific behavioural guidance in both information nudges to function as cues to action (*P* < 0.001).

Among statistically significant outcomes, AMDs ranged from 0.17 to 0.30 points on a five-point scale. Although the absolute magnitude should be interpreted in the context of the outcome scale and is not directly comparable across studies using different instruments, randomised evaluations of low-intensity information nudge have likewise generally reported modest, but statistically detectable changes in behavioural or psychological outcomes [34,35]. Moreover, because information nudges are inexpensive and readily scalable, even modest individual-level effects may translate into meaningful population-level benefits when implemented broadly [36,37].

Predictive modelling identified pre-existing health belief scores as the primary negative predictors of nudge responsiveness, indicating a cognitive ceiling effect, whereby individuals with lower baseline risk appraisals exhibited greater capacity for psychological shifts, while those with high baseline beliefs were less responsive [38–40]. Additionally, residing outside regions with prior NiV outbreaks promoted responsiveness across both perceived severity and benefits, possibly reflecting this aforementioned ceiling effect, as baseline perceived severity was significantly higher within regions with prior NiV outbreaks in our cohort. Finally, as both high (>5 times/day) and low (≤5 times/day) daily close contacts positively predicted increased perceived benefits, it is likely that this variable lacked discriminative predictive value. These findings could help guide targeted resource allocation to complement broader national communication efforts. The pronounced ceiling effect in regions with prior NiV outbreaks, coupled with the higher responsiveness of populations outside regions with prior NiV outbreaks, suggests that information-based nudges may be more effective in low-incidence settings. Early implementation of information-based nudges in these regions prior to widespread transmission may maximise cognitive shifts and improve preventive readiness [41]. Factors such as regional differences in media exposure, baseline risk perception, and varying levels of trust in health authorities might also contribute to the higher responsiveness observed outside outbreak regions [42,43]. Beyond these general predictors, frequent raw date palm sap consumption positively contributed to increased perceived severity, highlighting the necessity of culturally tailored health communication. Developing highly specific and localised messaging that directly addresses known high-risk dietary practices may be more effective at improving threat appraisal than generic warnings [44,45]. As deploying complex machine learning approaches at the primary care level is impractical, the identified predictors from the predictive modelling may provide useful insights into factors associated with responsiveness to information nudges.

While mediation analyses clarified the psychological pathways linking information nudges to behavioural intentions, we note that these intentions reflect responses to hypothetical scenarios, as no licensed NiV vaccines or therapeutics currently exist. The perceived severity nudge indirectly increased treatment intention by enhancing perceived severity, aligning with prior evidence that heightened perceptions of disease lethality strengthen motivation to adopt treatment-related actions [46]. Conversely, the perceived benefits nudge indirectly increased both vaccination and treatment intentions *via* cues to action, consistent with evidence that cues to action serve as critical stimuli facilitating the uptake of both preventive and therapeutic measures [47–49]. The remaining health belief dimensions did not mediate the effects of these information nudges on vaccination and treatment intention.

Regarding intention to promptly seek medical care, none of the health belief variables served as mediators, while the direct effects of the different framing nudges on this outcome were also non-significant (Figure S3 in the **Online Supplementary Document**). One possible explanation for this is that the actual decision to visit a healthcare facility could be constrained by complex unmeasured structural factors, including healthcare accessibility, financial costs, and the availability of local medical resources [50–52]. Briefly altering psychological health beliefs through text messages might, therefore, be insufficient to overcome these potential systemic constraints. Furthermore, perceived barriers, particularly the fear of social stigma captured in our survey, could impede the intention to seek care [52,53]. Our information nudges were not designed to reduce perceived barriers and did not significantly alter this dimension (**Table 2**). However, participants who did not intend to seek medical care exhibited significantly higher perceived barrier scores than those willing to seek care across both pre- and post-nudge assessments. This indicates a potential role for perceived barriers in shaping behavioural intentions that should be explored in future research. Identifying these distinct pathways is critical for optimising health communication design. Public health strategies can be refined by deploying severity-focused messaging to drive the acceptance of therapeutics, while leveraging explicit information on the benefits of protective measures as actionable cues to mobilise vaccination and treatment readiness. Given the mediating role of cues to action, communication materials should incorporate explicit behavioural triggers, including specific guidance to avoid unboiled date palm sap, thoroughly wash fruit, and wear masks during patient care.

Our study provides empirical data on population-level health beliefs and protective behavioural intentions regarding the NiV in India. Its results suggest that integrating targeted communication strategies, including prioritising low-incidence areas, tailoring messages for high-risk behaviours, and designing path-specific nudges, could help inform future public health campaigns and foster greater population-level readiness for NiV outbreaks.

The strength of this study lies in its novelty as one of the first large-scale surveys to assess population-level knowledge and health beliefs regarding the NiV in India. The integration of an experimental survey design with predictive modelling and mediation analysis further provides a framework for investigating the potential psychological mechanisms driving protective behaviours against NiV. However, several limitations must be noted. First, our survey was conducted *via* an online platform, which introduced selection bias, as web panel participants often possess greater digital access and health literacy than the general public [54], which is best seen in our sample’s disproportionately high rates of tertiary education (88.7%) and urban residence (74.9%). Consequently, these findings may not be fully generalisable to rural, lower-income, or lower-literacy populations. Similarly, the relative importance of predictors identified in our models should be viewed as exploratory and may not be directly generalisable across different populations or contexts. Furthermore, while we oversampled Kerala and West Bengal to capture high-risk populations, we did not apply post-stratification survey weights. Although we adjusted for residence in prior outbreak regions to reduce potential confounding, the unadjusted national estimates may still be subject to oversampling bias. Future studies employing nationally representative sampling and weighting approaches may, therefore, help assess the generalisability of our findings. Second, we relied on self-reported data to assess psychological dimensions and behavioural intentions based on hypothetical scenarios. Because no licensed Nipah vaccines or specific therapeutics currently exist, participants were inherently responding to countermeasures that are still under development. This may have introduced bias and affected the validity of the reported intentions, as the measured intentions for vaccination and treatment may not perfectly reflect actual uptake during a real-world outbreak. Future research should incorporate objective behavioural tracking to verify whether these nudge-induced cognitive shifts translate into tangible compliance. Third, we did not pilot test the survey prior to full deployment or validate it in a similar manner. Fourth, we assessed each HBM construct using a single-item measure to reduce respondent burden in a rapid web-based survey, which precludes formal reliability assessment and may not fully capture construct complexity. Finally, we evaluated cognitive responses immediately after exposure to the information nudges, which limits our ability to draw causal inferences from the mediation analyses. The long-term durability of these psychological shifts remains unknown. It is possible that the immediate increases in perceived severity and cues to action might attenuate over time, suggesting that future longitudinal studies are necessary to determine the optimal frequency of sustained risk communication during outbreaks.

## CONCLUSIONS

Our findings demonstrate that targeted information nudges effectively alter health beliefs regarding the NiV. The perceived severity nudge applied in our survey increased both perceived severity and cues to action, while the perceived benefits nudge enhanced cues to action. Predictive modelling identified individuals with lower baseline perceived severity and benefits, populations residing outside regions with prior NiV outbreaks, and frequent consumers of raw date palm sap as responsive to these information nudges, while mediation analyses indicated that these altered cognitions form distinct psychological pathways. The perceived severity nudge indirectly increased treatment intention through perceived severity, while the perceived benefits nudge indirectly increased both vaccination and treatment intentions *via* cues to action. These findings suggest that policymakers could incorporate localised communication strategies targeting these specific pathways alongside broader national efforts to enhance population-level preparedness for future NiV outbreaks.

## Additional material


Online Supplementary Document

## Data Availability

**Data availability:** Data can be obtained from the corresponding author.
