# Supplementary material for: Information nudging, attitude, and protective behaviours against Nipah virus in India, 2026: a national experimental survey
Source: J Glob Health. 2026 Nov 6;16:04362. doi: 10.7189/jogh.16.04362 (PMC13741164; doi:10.7189/jogh.16.04362)
Supplement: Supplementary file 1 [file jogh-16-04362-s001.pdf]

Supplement to: Wang S, Zhou L, Xing K, Zhang Z, Wu S, Liu Q, Liu M, Zhang H, Kota SH, Tao L, Liu J. Information nudging, attitude, and protective behaviours against Nipah virus in India, 2026: a national experimental survey. J Glob Health. 2026;16:04362.

Survey questionnaire 2

Supplementary Methods: Data Processing and Variable Definitions 24

Figure S1 Study profile. 27

Figure S2 ROC curves of six predictive models for identifying natural susceptibility to cognitive changes. 28

Figure S3 Mediation pathways of information nudges on promptly seeking medical care. 29

Table S1 Predictive performance of machine learning models and logistic regression for identifying susceptibility to cognitive changes. 30

Table S2 Mediation effects of severity- and benefit-focused information nudges on vaccination and treatment intentions. 31

Table S3 Sensitivity analysis of predictive models using a minimal feature set 35

Table S4 Sensitivity analysis of mediation models using a minimal covariate adjustment set 36

Table S5 Sensitivity analysis of mediation models using subjective cognitive proxies. 39

Table S6 Sensitivity analysis of mediation models using generalised high-risk exposure histories. 42

STROBE Statement—Checklist of items that should be included in reports of *cross-sectional studies* 45

Survey questionnaire

Version 1 (English)

Public Knowledge and Behavioral Survey on Nipah Virus

Dear Participant,
This study aims to understand the public's knowledge of Nipah virus (NiV) and preventive/protective behaviors. Participation is voluntary. The questionnaire is anonymous and there are no right or wrong answers. All information you provide will be kept strictly confidential and used only for academic research purposes.
Thank you!
Public Knowledge and Behavioral Survey on Nipah Virus Research Group

Q1. Do you agree to participate in this survey? *(Single choice) **

○ Yes, I agree to participate in this survey. *(Continue the survey)*

○ No, I do not agree to participate in this survey. *(End the survey)*

Q2. Which state do you currently reside in (long-term)? *(Single choice) **

○ West Bengal

○ Kerala

○ Andaman and Nicobar Islands

○ Andhra Pradesh

○ Arunachal Pradesh

○ Assam

○ Bihar

○ Chandigarh

○ Chhattisgarh

○ Dadra and Nagar Haveli

○ Daman and Diu

- Delhi
- Goa
- Gujarat
- Haryana
- Himachal Pradesh
- Jammu and Kashmir
- Jharkhand
- Karnataka
- Lakshadweep
- Madhya Pradesh
- Maharashtra
- Manipur
- Meghalaya
- Mizoram
- Nagaland
- Odisha
- Puducherry
- Punjab
- Rajasthan
- Sikkim
- Tamil Nadu
- Tripura
- Uttar Pradesh

○ Uttarakhand

○ Telangana

Q3. What type of residential area do you live in? *(Single choice)* *

○ Rural
○ Urban

Q4. What is your gender? *(Single choice)* *
○ Male
○ Female
○ Third gender / transgender / prefer not to disclose

Q5. What is your age? ____ years *(integer)**

Q6. What is your highest level of education? *(Single choice)**
○ Primary school or below
○ Secondary school
○ Junior college / Bachelor's degree
○ Master's degree or above

Q7. What is your occupation? *(Single choice)**
○ Healthcare worker
○ Veterinarian / animal-related professional
○ Livestock farming / breeding / slaughtering / meat processing-related
○ Student
○ Company employee / service industry worker
○ Unemployed / homemaker / caregiver
○ Other

Q8. Do any of the family members living with you fall into the following categories? *(Multiple choice)**
□ Children (<18 years)
□ Older adults (≥60 years)
□ Pregnant women
□ People with weakened immunity / chronic diseases
□ None of the above

Q9. Have you ever heard of Nipah virus (NiV) / Nipah virus infection? *(Single choice)**
○ Never heard of it

○ Have heard of it but do not know much
○ Have heard of it and know a little
○ Know it fairly well

Q10. In the past 12 months, have you seen or heard any information related to Nipah? *(Single choice)**
○ No
○ Yes (once)
○ Yes (multiple times)
○ Not sure

Q11. What are your most commonly used sources for infectious disease/health information? *(Multiple choice; select up to 2)**
□ Health department/government announcements
□ Doctors/healthcare institutions
□ Television/radio/newspapers
□ Schools/community organizations
□ Social media (e.g., WhatsApp, Facebook)
□ Family and friends
□ Other

Q12. Which source do you trust the most? *(Single choice)**
○ Health department/government announcements
○ Doctors/healthcare institutions
○ Television/radio/newspapers
○ Social media
○ Family/friends/community
○ Other / Not sure

Q13. Please judge whether the following statements about Nipah virus are correct. *(Matrix question)**

Items	1.True	2. False	3. Not sure
**Q13-1.** Nipah virus infection may be related to bat exposure (e.g., food contaminated by bats).	□	□	□
**Q13-2.** Nipah virus can be transmitted through close contact/person-to-person caregiving.	□	□	□
**Q13-3.** Nipah virus infection may cause fever and respiratory symptoms.	□	□	□
**Q13-4.** Severe Nipah virus infection may cause neurological symptoms such as altered consciousness.	□	□	□

**Q13-5.** There are currently widely available specific medicines that can "cure" Nipah virus infection in most cases.	□	□	□
**Q13-6.** Seeking medical care promptly when severe symptoms occur can help reduce health risks.	□	□	□

Q14. In the past 3 months, have you experienced any of the following? (Select all that apply.) *(Multiple choice)**

□ Provided close care to a family member with fever/obvious respiratory symptoms
□ Frequently attended crowded gatherings (festivals/meetings, etc.)
□ Had contact with sick/dead animals or animal secretions
□ None of the above

Q15. In the past 12 months, have you worked in or frequently been exposed to any of the following settings? (Select all that apply.) *(Multiple choice)**

□ Livestock farming/breeding facilities
□ Slaughtering/meat processing
□ Contact with/capture/handling of wild animals
□ None of the above

Q16. Please indicate the extent to which you agree or disagree with the following statements.
(1 = Strongly disagree, 2 = Disagree, 3 = Not sure, 4 = Agree, 5 = Strongly agree)
*(Matrix question)**

Q16-1. I think I may be at risk of being infected with Nipah virus.
Q16-2. I think that if I were infected with Nipah virus, it could lead to serious health consequences (e.g., hospitalization, death).
Q16-3. I think that taking protective measures recommended by the health authorities (e.g., washing hands frequently, avoiding contact with fruit bats) can effectively reduce the risk of Nipah virus infection.
Q16-4. I worry that if I seek medical care for suspected Nipah symptoms or talk about it, I may face stigma or negative comments from others.
Q16-5. I am confident that I can correctly follow guidance to carry out key protective measures against Nipah virus (e.g., proper handwashing, food hygiene).
Q16-6. Recently, I have received clear guidance on how to prevent Nipah virus infection from the media, community campaigns, or healthcare workers.

Q17. If a family member develops severe symptoms such as "high fever + altered consciousness / severe breathing difficulty," what would you be most likely to do? *(Single choice)**

○ Seek medical care immediately
○ Seek medical care within 24 hours
○ Seek medical care within 1–2 days
○ Seek medical care after 3 days
○ Consult a pharmacy/community doctor first, then decide whether to go to the hospital
○ Prefer not to seek medical care / manage it on my own
○ Not sure

Q18. How often do you usually handle raw pork? (Handling includes buying raw pork or cooking with it.) *(Single choice)**
○ Almost every day
○ 2–6 days per week
○ 1 day per week
○ 1–3 times per month
○ Rarely / never

Q19. How often do you usually drink raw date palm sap (unboiled/unprocessed)? *(Single choice)**
○ Almost every day
○ 2–6 days per week
○ 1 day per week
○ 1–3 times per month
○ Rarely / never

Q20. In the past 7 days, about how many close contacts with people who do not live with you did you have on average per day? [Definition of close contact: Face-to-face interaction within **1 meter**, or being in a confined space together for **≥15 minutes**, or having **direct physical contact** (e.g., handshaking, hugging, caregiving). Brief pass-by encounters are **not** included.] *(Single choice)**
○ 0 times/day (Almost no close contact with non-household members)
○ 1–2 times/day
○ 3–5 times/day
○ 6–10 times/day
○ 11–20 times/day
○ ≥21 times/day
○ Not sure

Please read the following information (30 seconds):

Information A:

According to the World Health Organization, Nipah virus infection is an infectious disease that can be transmitted from animals to humans. It can also spread through contaminated food (e.g., unboiled raw date palm sap or fruit contaminated by bats) and through close person-to-person contact (especially when caring for a patient). In past outbreaks, the case-fatality rate was approximately 40%–75%. Common symptoms after infection include fever, headache, muscle pain, vomiting, and sore throat; respiratory symptoms such as cough and difficulty breathing may also occur. In severe cases, confusion, drowsiness, seizures/encephalitis, and even coma may occur.

Drinking unboiled date palm sap, eating unwashed fruit, or lacking protective measures while caring for patients can increase the risk of infection and transmission to family members. If high fever is accompanied by difficulty breathing or altered consciousness, please seek medical care as soon as possible. In daily life, reduce unnecessary close contact, wash hands frequently, and use appropriate protection when providing care.

Information B:

According to the World Health Organization, Nipah virus infection is an infectious disease that can be transmitted from animals to humans. It can also spread through contaminated food (e.g., unboiled raw date palm sap or fruit contaminated by bats) and through close person-to-person contact (especially when caring for a patient). In past outbreaks, the case-fatality rate has been approximately 40%–75%. Common symptoms include fever, headache, muscle pain, vomiting, and sore throat; respiratory symptoms such as cough and difficulty breathing may also occur. In severe cases, confusion, drowsiness, seizures/encephalitis, and even coma may occur.

Avoiding unboiled date palm sap, thoroughly washing and peeling fruit before eating, and wearing a mask and washing hands frequently when caring for a patient can reduce the risk of infection and transmission, helping protect you and your family. If you develop a high fever accompanied by difficulty breathing or altered consciousness, seek medical care as soon as possible and inform the clinician about any recent exposures and dietary history to help ensure timely and appropriate management and to reduce further spread.

Information C:

Please take a 30-second break before answering the next question.

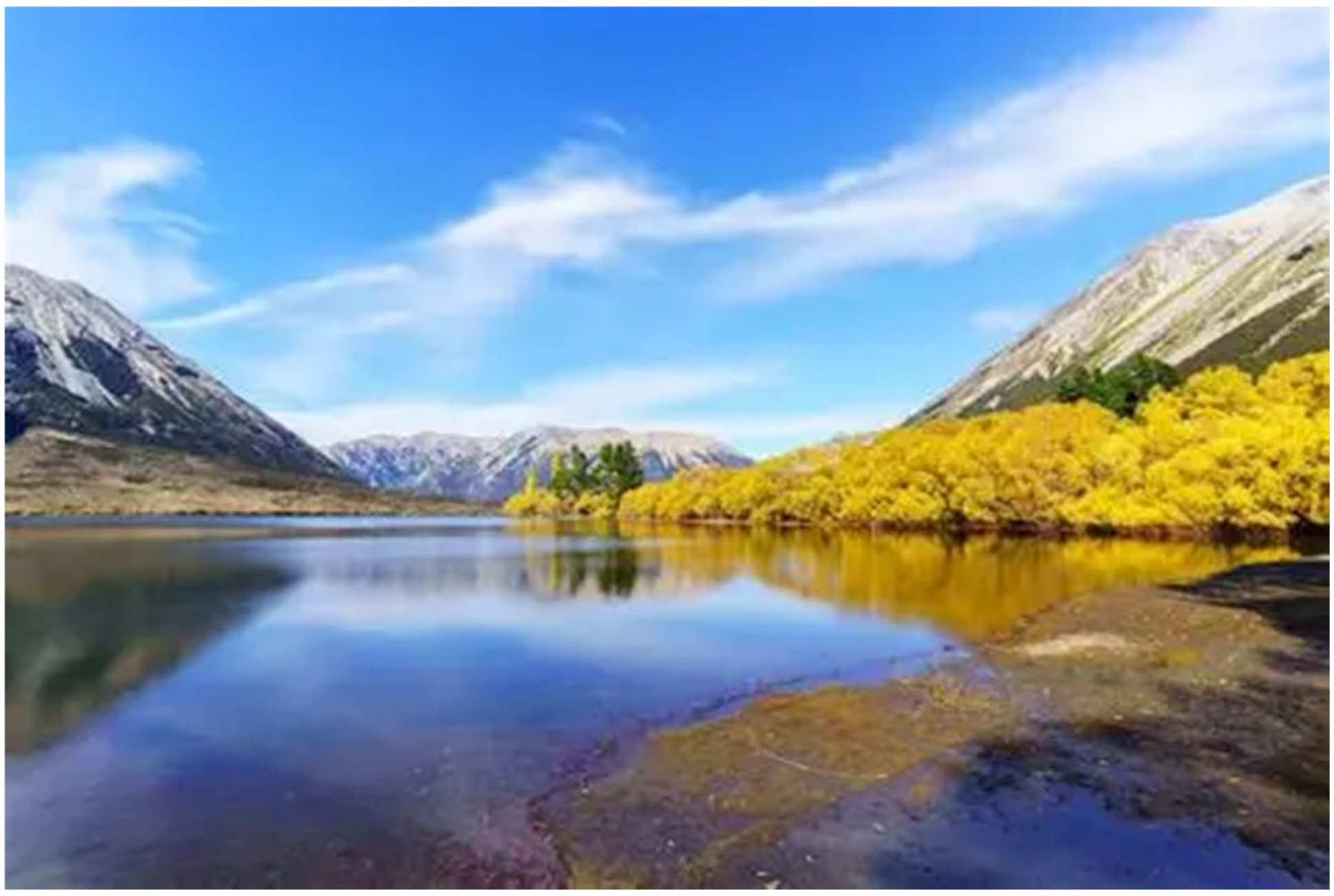

Q21. Please indicate the extent to which you agree or disagree with the following statements.
(1 = Strongly disagree, 2 = Disagree, 3 = Not sure, 4 = Agree, 5 = Strongly agree)
*(Matrix question)**

Q21-1. I think I may be at risk of being infected with Nipah virus.
Q21-2. I think that if I were infected with Nipah virus, it could lead to serious health consequences (e.g., hospitalization, death).
Q21-3. I think that taking protective measures recommended by the health authorities (e.g., washing hands frequently, avoiding contact with fruit bats) can effectively reduce the risk of Nipah virus infection.
Q21-4. I worry that if I seek medical care for suspected Nipah symptoms or talk about it, I may face stigma or negative comments from others.
Q21-5. I am confident that I can correctly follow guidance to carry out key protective measures against Nipah virus (e.g., proper handwashing, food hygiene).
Q21-6. Recently, I have received clear guidance on how to prevent Nipah virus infection from the media, community campaigns, or healthcare workers.

Q22. If there is an official announcement/case report of Nipah virus in your area, which of the following measures would you be willing to take? (Select all that apply.) *(Multiple choice) **

□ Reduce gatherings / avoid crowded places
□ Seek medical care promptly if symptoms occur and report any exposure history

□ Wear a mask and wash hands frequently when caring for others
□ Prioritize information from official sources
□ None of the above / Not sure

Q23. If a safe and effective Nipah vaccine becomes available in the future, would you be willing to get vaccinated? *(Single choice)* *

○ Very willing
○ Willing
○ Not sure
○ Unwilling
○ Very unwilling

Q24. If a safe and effective treatment for Nipah becomes available in the future, would you be willing to use it? *(Single choice)* *

○ Very willing
○ Willing
○ Not sure
○ Unwilling
○ Very unwilling

End of the questionnaire. Thank you for your participation!

Version 2 (Hindi)

निपाह वायरस पर लोगों की जानकारी और व्यवहार सर्वेक्षण

प्रिय प्रतिभागी,

इस स्टडी का उद्देश्य यह समझना है कि लोगों को निपाह वायरस (NiV) के बारे में कितनी जानकारी है और वे बचाव/सुरक्षात्मक व्यवहार कैसे करते हैं। इसमें हिस्सा लेना स्वैच्छिक है। ये सर्वेक्षण गुमनाम है और इसमें कोई सही या गलत जवाब नहीं हैं। आपके द्वारा दी गई सभी जानकारी को पूरी तरह से गोपनीय रखा जाएगा और सिर्फ़ अकादमिक रिसर्च के उद्देश्यों के लिए इस्तेमाल की जाएगी।

धन्यवाद!

निपाह वायरस पर लोगों की जानकारी और व्यवहार सर्वेक्षण रिसर्च ग्रुप

Q1. क्या आप इस सर्वेक्षण में भाग लेने के लिए सहमत हैं? *(एक विकल्प) **

○ हां, मैं इस सर्वेक्षण में भाग लेने के लिए सहमत हूं। *(सर्वेक्षण जारी रखें)*

○ नहीं, मैं इस सर्वेक्षण में भाग लेने के लिए सहमत नहीं हूं। *(सर्वे समाप्त करें)*

Q2. आप अभी किस राज्य में रहते हैं (लंबे समय से)? *(एक विकल्प) **

○ पश्चिम बंगाल

○ केरल

○ अंडमान और निकोबार द्वीप समूह

○ आंध्र प्रदेश

○ अरुणाचल प्रदेश

○ असम

○ बिहार

○ चंडीगढ़

○ छत्तीसगढ़

○ दादरा और नगर हवेली

○ दमन और दीव

○ दिल्ली

○ गोवा

○ गुजरात

○ हरियाणा

○ हिमाचल प्रदेश

○ जम्मू और कश्मीर

○ झारखंड

○ कर्नाटक

- लक्षद्वीप
- मध्य प्रदेश
- महाराष्ट्र
- मणिपुर
- मेघालय
- मिज़ोरम
- नागालैंड
- ओडिशा
- पुदुचेरी
- पंजाब
- राजस्थान
- सिक्किम
- तमिलनाडु
- त्रिपुरा
- उत्तर प्रदेश

○ उत्तराखंड

○ तेलंगाना

Q3. आप किस प्रकार के आवासीय क्षेत्र में रहते हैं? *(एक विकल्प)* *

○ ग्रामीण

○ शहरी

Q4. आपका लिंग क्या है? *(एक विकल्प)* *

○ पुरुष

○ महिला

○ तीसरा लिंग / ट्रांसजेंडर / बताना नहीं चाहते

Q5. आपकी उम्र कितनी है? ____ साल *(पूर्णांक)* *

Q6. आपकी शिक्षा का उच्चतम स्तर क्या है? *(एक विकल्प)* *

○ प्राथमिक स्कूल या उससे कम

○ माध्यमिक विद्यालय

○ जूनियर कॉलेज / बैचलर की डिग्री

○ मास्टर्स डिग्री या इससे ज़्यादा

Q7. आपका पेशा क्या है? *(एक विकल्प)* *

○ स्वास्थ्य सेवा कार्यकर्ता

○ जानवरों के डॉक्टर हैं / जानवरों से जुड़ा पेशा

○ पशुपालन / पैदा करने / काटने / मांस तैयार करने से जुड़ा

○ स्टूडेंट

○ कंपनी में कर्मचारी हैं / सर्विस इंडस्ट्री वर्कर

○ बेरोज़गार / घर संभालते/संभालती हैं / किसी की देखभाल करते/करती हैं

○ अन्य

Q8. क्या आपके साथ रहने वाले परिवार के सदस्यों में से कोई निम्न कैटेगरियों में आता है? *(एक से ज़्यादा विकल्प)* *

□ बच्चे (<18 साल)

□ बुज़ुर्ग वयस्क (≥60 साल)

□ गर्भवती महिलाएँ

□ कमज़ोर इम्युनिटी / पुरानी बीमारियां

□ उपरोक्त में से कोई नहीं

Q9. क्या आपने कभी निपाह वायरस (NiV) / निपाह वायरस संक्रमण के बारे में सुना है? *(एक विकल्प)* *

○ इसके बारे में कभी नहीं सुना

○ इसके बारे में सुना है लेकिन ज़्यादा जानकारी नहीं है

○ इसके बारे में सुना है और थोड़ा जानता/जानती हूं

○ काफ़ी अच्छे से जानता/जानती हूं

Q10. पिछले 12 महीनों में, क्या आपने निपाह से संबंधित कोई जानकारी देखी या सुनी है? *(एक विकल्प)* *

○ नहीं

○ हां (एक बार)

○ हां (बहुत बार)

○ ठीक से पता नहीं है

Q11. संक्रामक रोग/स्वास्थ्य से जुड़ी जानकारी के लिए आप सबसे ज़्यादा किन सोर्सेस का इस्तेमाल करते हैं? *(एक से ज़्यादा विकल्प; ज़्यादा से ज़्यादा 2 चुनें)**

□ स्वास्थ्य विभाग/सरकारी घोषणाएं

□ डॉक्टर/स्वास्थ्य देखभाल संस्थान

□ टेलीविज़न/रेडियो/अखबार

□ स्कूल/समुदाय के संगठन

□ सोशल मीडिया (जैसे, व्हाट्सऐप, फ़ेसबुक)

□ परिवार और दोस्त

□ अन्य

Q12. आप किस सोर्स पर सबसे ज़्यादा भरोसा करते हैं? *(एक विकल्प)**

○ स्वास्थ्य विभाग/सरकारी घोषणाएं

○ डॉक्टर/स्वास्थ्य देखभाल संस्थान

○ टेलीविज़न/रेडियो/अखबार

○ सोशल मीडिया

○ परिवार/दोस्त/समुदाय

○ अन्य / ठीक से पता नहीं है

Q13. कृपया बताएं कि निपाह वायरस के बारे में नीचे दी गई बातें सही हैं या नहीं।

*(मैट्रिक्स सवाल)**

Items	1.सही	2. गलत	3. ठीक से पता नहीं है
**Q13-1.** निपाह वायरस का संक्रमण चमगादड़ के संपर्क में आने से हो सकता है (जैसे, चमगादड़ों से दूषित खाना)।	□	□	□
**Q13-2.** निपाह वायरस नज़दीकी संपर्क/व्यक्ति-दर-व्यक्ति देखभाल के ज़रिये फ़ैल सकता है।	□	□	□
**Q13-3.** निपाह वायरस होने पर बुखार और सांस की तकलीफ़ें हो सकती हैं.	□	□	□
**Q13-4.** गंभीर निपाह वायरस के संक्रमण से दिमाग पर असर हो सकता है जैसे बेहोशी।	□	□	□
**Q13-5.** अभी ऐसी खास दवाएं आसानी से मिल रही हैं जो ज़्यादातर मामलों में निपाह वायरस के संक्रमण को "ठीक कर सकती हैं"।	□	□	□
**Q13-6.** गंभीर लक्षण होने पर जल्दी डॉक्टर के पास जाना सेहत के जोखिम कम कर सकता है।	□	□	□

Q14. पिछले 3 महीनों में, क्या आपने निम्न में से किसी का अनुभव किया है? (लागू होने वाले सभी को चुनें।) *(एक से ज़्यादा विकल्प)**

□ परिवार के किसी सदस्य की नज़दीकी देखभाल की जिसे बुखार/साफ़ दिखने वाली सांस की तकलीफ़ें थीं

□ अक्सर भीड़भाड़ वाले समारोहों (त्यौहार/मीटिंग्स, आदि) में शामिल हुए

□ बीमार/मरे हुए जानवरों या जानवरों से निकलने वाली चीज़ों के संपर्क में आए थे

□ उपरोक्त में से कोई नहीं

Q15. पिछले 12 महीनों में, क्या आप इनमें से किसी जगह पर काम कर रहे थे या बार-बार जाते थे? (लागू होने वाले सभी को चुनें।) *(एक से ज़्यादा विकल्प)**

□ पशुपालन/ब्रीडिंग फ़ैसिलिटीज़

□ जानवर काटना/मांस तैयार करना

□ जंगली जानवरों को छूना/पकड़ना/हाथ लगाना

□ उपरोक्त में से कोई नहीं

Q16. कृपया ये बताएं कि आप निम्न कथनों से किस हद तक सहमत या असहमत हैं।

(1 = दृढ़ता से असहमत, 2 = असहमत, 3 = निश्चित नहीं, 4 = सहमत, 5 = दृढ़ता से सहमत)

*(मैट्रिक्स सवाल)**

Q16-1. मुझे लगता है कि मुझे निपाह वायरस से संक्रमित होने का खतरा हो सकता है।

Q16-2. मुझे लगता है कि अगर मैं निपाह वायरस से संक्रमित हो जाऊं, तो इसके गंभीर स्वास्थ्य परिणाम हो सकते हैं (जैसे, अस्पताल में भर्ती होना, मौत)।

Q16-3. मुझे लगता है कि स्वास्थ्य विभाग के बताए गए बचाव के तरीके अपनाने से (जैसे, बार-बार हाथ धोना, फ़ल खाने वाले चमगादड़ों से दूर रहना) निपाह वायरस का संक्रमण होने का खतरा असरदार तरीके से कम किया जा सकता है।

Q16-4. मुझे डर लगता है कि अगर मैं निपाह होने की आशंका वाले लक्षणों के लिए डॉक्टर के पास जाऊं या इसके बारे में बात करूं, तो लोग मेऋ बदनामी कर सकते हैं या मुझे बुरा बर्ताव का सामना करना पड़ सकता है।

Q16-5. मुझे विश्वास है कि मैं निपाह वायरस के खिलाफ़ प्रमुख सुरक्षात्मक उपायों को करने के लिए दिशा-निर्देशों का सही ढंग से पालन कर सकता/सकती हूं (जैसे, सही तरीके से हाथ धोना, खाने की स्वच्छता)।

Q16-6. हाल ही में, मुझे मीडिया, कम्युनिटी कैंपेनों, या स्वास्थ्य कर्मियों से निपाह वायरस से बचने की साफ़ जानकारी मिली है।

Q17. अगर परिवार के किसी सदस्य में गंभीर लक्षण जैसे "तेज़ बुखार + बेहोशी जैसा / सांस लेने में बहुत तकलीफ़" होने लगें, तो आप सबसे ज़्यादा क्या करने की सोचेंगे? *(एक विकल्प)* *

○ तुरंत इलाज करवाना

○ 24 घंटे के अंदर इलाज करवाना

○ 1-2 दिनों के अंदर इलाज करवाना

○3 दिनों के बाद इलाज करवाना

○ पहले फ़ार्मेसी/कम्युनिटी डॉक्टर से परामर्श करें, फ़िर अस्पताल जाने के बारे में फ़ैसला लें

○ इलाज नहीं कराऊंगा/कराऊंगी / खुद ही देख लूंगा/लूंगी

○ ठीक से पता नहीं है

Q18. आप आम तौर पर कितनी बार कच्चे सुअर के मांस को छूते/छूती हैं? (छूने में कच्चे सुअर के मांस को खरीदना या इससे खाना पकाना शामिल है।) *(एक विकल्प)* *

○ लगभग रोज़

○ हफ़्ते में 2-6 दिन

○ हर हफ्ते 1 दिन

○ महीने में 1-3 बार

○ शायद ही कभी / कभी नहीं

Q19. आप आमतौर पर कितनी बार कच्चे खजूर का रस (बिना उबला/बिना प्रोसेस किया हुआ) पीते हैं? *(एक विकल्प)* *

○ लगभग रोज़

○ हफ़्ते में 2-6 दिन

○ हर हफ्ते 1 दिन

○ महीने में 1-3 बार

○ शायद ही कभी / कभी नहीं

Q20. पिछले 7 दिनों में, उन लोगों के साथ जो आपके साथ नहीं रहते हैं, आपके औसतन प्रति दिन कितने नज़दीकी संपर्क हुए? [नज़दीकी संपर्क से तात्पर्य है: **1 मीटर** की दूरी के अंदर आमने-सामने बातचीत, या ≥**15 मिनट** तक एक सीमित जगह पर एक साथ रहना, या **सीधा शारीरिक संपर्क** होना (जैसे हाथ मिलाना, गले लगाना, देखभाल करना)। छोटी मुलाकातें शामिल **नहीं** हैं।] *(एक विकल्प)* *

○ **0 बार/दिन (गैर-घरेलू सदस्यों के साथ लगभग कोई नज़दीकी संपर्क नहीं)**

○ **1–2 बार/दिन**

○ **3–5 बार/दिन**

○ **6–10 बार/दिन**

○ **11–20 बार/दिन**

○ **≥21 बार/दिन**

○ **ठीक से पता नहीं है**

कृपया निम्नलिखित जानकारी पढ़ें (30 सेकंड):

जानकारी A:

विश्व स्वास्थ्य संगठन के अनुसार, निपाह वायरस का संक्रमण एक संक्रामक बीमारी है जो जानवरों से इंसानों में फ़ैल सकती है। यह दूषित खाने से भी फ़ैल सकती है (जैसे, बिना उबाला गया कच्चा खजूर का रस या चमगादड़ों द्वारा गंदे हुए फ़ल) और लोगों के बीच नज़दीकी संपर्क से भी (खासकर जब किसी मरीज़ की देखभाल कर रहे हों)। पिछली महामारियों में, मृत्यु की दर लगभग 40%–75% थी। संक्रमण के बाद सामान्य लक्षणों में बुखार, सिरदर्द, मांसपेशियों में दर्द, उल्टी और गले में खराश शामिल हैं; सांस से जुडी तकलीफ़ें जैसे खांसी और सांस लेने में दिक्कत भी हो सकती है। गंभीर मामलों में, समझ में गड़बड़ी, बेहोशी, बहुत नींद आना, दौरे/दिमागी बुखार और यहां तक कि कोमा भी हो सकता है।

बिना उबले खजूर काे रस पीना, बिना धुले फ़ल खाना, या मरीज़ों की देखभाल करते समय बचाव के उपाय न करना संक्रमण होने और परिवार के सदस्यों में फ़ैलने का खतरा बढ़ा सकते हैं। अगर आपको तेज़ बुखार के साथ सांस लेने में तकलीफ़ या बेहोशी जैसा हो, तो कृपया जितनी जल्दी हो सके डॉक्टर के पास जाएंं। रोज़ाना की ज़िंदगी में, ज़रूरत न हो तो नज़दीकी संपर्क कम करें, बार-बार हाथ धोएं, और देखभाल करते समय सही बचाव के उपाय का इस्तेमाल करें।

जानकारी B:

विश्व स्वास्थ्य संगठन के अनुसार, निपाह वायरस का संक्रमण एक संक्रामक बीमारी है जो जानवरों से इंसानों में फ़ैल सकती है। यह दूषित खाने से भी फ़ैल सकती है (जैसे, बिना उबाला गया कच्चा खजूर का रस या चमगादड़ों द्वारा गंदे हुए फ़ल) और लोगों के बीच नज़दीकी संपर्क से भी (खासकर जब किसी मरीज़ की देखभाल कर रहे हों)। पिछली महामारियों में, मृत्यु की दर लगभग 40%–75% रही है। सामान्य लक्षणों में बुखार, सिरदर्द, मांसपेशियों में दर्द, उल्टी, और गले में खराश शामिल है। सांस से जुड़ी तकलीफ़ें जैसे खांसी और सांस लेने में दिक्कत भी हो सकती है। गंभीर मामलों में, समझ में गड़बड़ी, बेहोशी, बहुत नींद आना, दौरे/दिमागी बुखार, और यहां तक कि कोमा भी हो सकता है।

बिना उबाले गए खजूर के रस से बचना, फ़ल खाने से पहले अच्छे से धोना और छीलना, और मरीज़ों की देखभाल करते समय मास्क पहनना और बार-बार हाथ धोना संक्रमण और फ़ैलाव का खतरा कम कर सकता है, जो आपको और आपके परिवार को सुरक्षित रखने में मदद करता है। अगर आपको तेज़ बुखार के साथ सांस लेने में तकलीफ़ या बेहोशी जैसा हो, तो जितनी जल्दी हो सके डॉक्टर के पास जाएं और डॉक्टर को हाल ही में हुए किसी भी संपर्क और खाने की जानकारी दें ताकि सही समय पर सही इलाज हो सके और आगे फ़ैलाव रुक सके।

जानकारी C:

कृपया अगले सवाल का जवाब देने से पहले 30-सेकंड का ब्रेक लें।

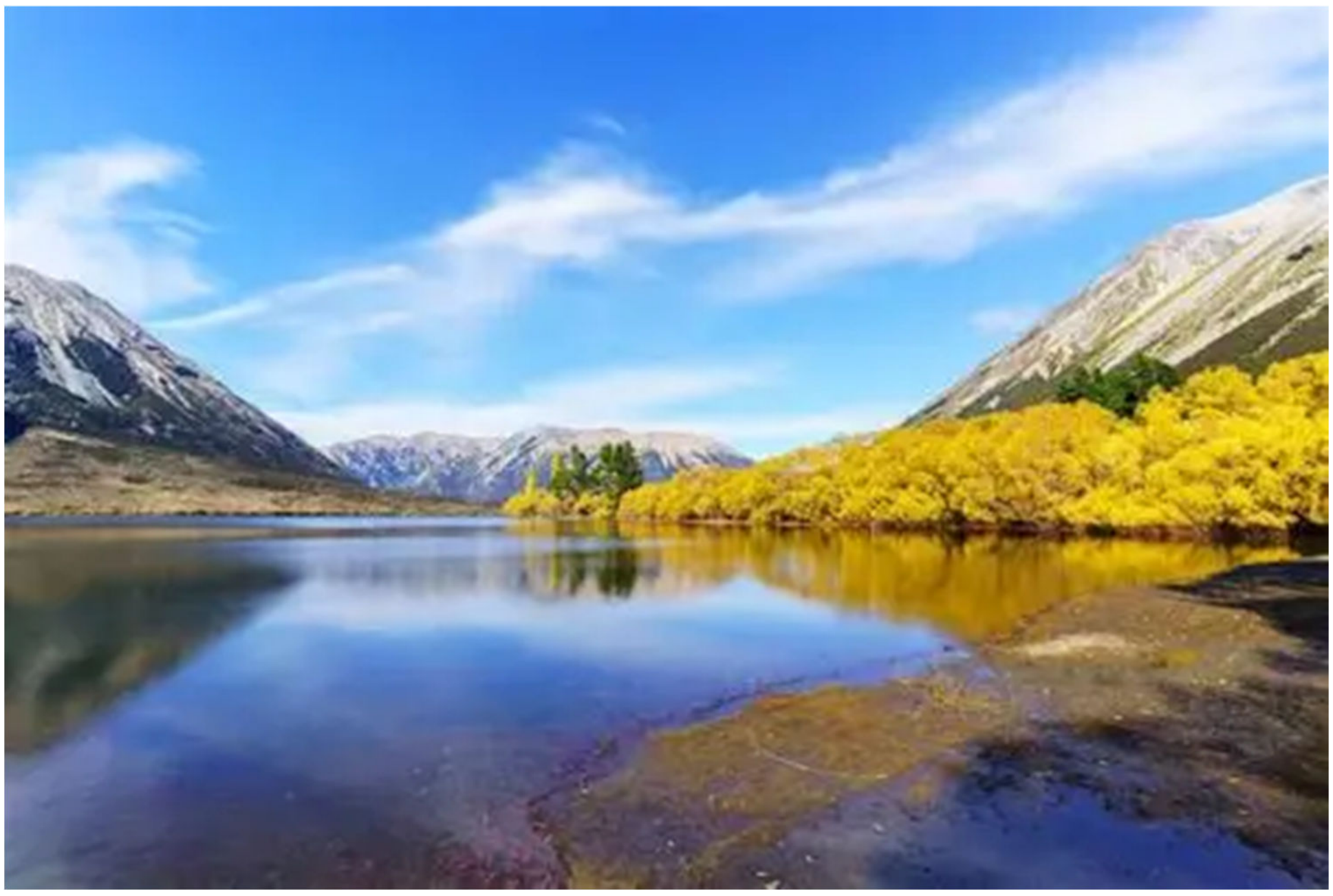

Q21. कृपया ये बताएं कि आप निम्न कथनों से किस हद तक सहमत या असहमत हैं।

(1 = दृढ़ता से असहमत, 2 = असहमत, 3 = निश्चित नहीं, 4 = सहमत, 5 = दृढ़ता से सहमत)

(मैट्रिक्स सवाल) *

Q21-1. मुझे लगता है कि मुझे निपाह वायरस से संक्रमित होने का खतरा हो सकता है।

Q21-2. मुझे लगता है कि अगर मैं निपाह वायरस से संक्रमित हो जाऊं, तो इसके गंभीर स्वास्थ्य परिणाम हो सकते हैं (जैसे, अस्पताल में भर्ती होना, मौत)।

Q21-3. मुझे लगता है कि स्वास्थ्य विभाग के बताए गए बचाव के तरीके अपनाने से (जैसे, बार-बार हाथ धोना, फ़ल खाने वाले चमगादड़ों से दूर रहना) निपाह वायरस का संक्रमण होने का खतरा असरदार तरीके से कम किया जा सकता है।

Q21-4. मुझे डर लगता है कि अगर मैं निपाह होने की आशंका वाले लक्षणों के लिए डॉक्टर के पास जाऊं या इसके बारे में बात करूं, तो लोग मेरी बदनामी कर सकते हैं या मुझे बुरा बर्ताव

का सामना करना पड़ सकता है।

Q21-5. मुझे विश्वास है कि मैं निपाह वायरस के खिलाफ़ प्रमुख सुरक्षात्मक उपायों को करने के लिए दिशा-निर्देशों का सही ढंग से पालन कर सकता/सकती हूं (जैसे, सही तरीके से हाथ धोना, खाने की स्वच्छता)।

Q21-6. हाल ही में, मुझे मीडिया, कम्युनिटी कैंपेनों, या स्वास्थ्य कर्मियों से निपाह वायरस से बचने की साफ़ जानकारी मिली है।

Q22. अगर आपके इलाके में निपाह वायरस की कोई सरकारी खबर आए/केस मिले, तो आप निम्न में से कौन से उपाय अपनाएंगे? (लागू होने वाले सभी को चुनें।) *(एक से ज़्यादा विकल्प) **

□ जमावड़े कम करना / भीड़-भाड़ वाली जगहों से दूर रहना

□ लक्षण आने पर तुरंत इलाज लेना और बताना कि कहां संपर्क हुआ था

□ दूसरों की देखभाल करते समय मास्क पहनना और हाथ बार-बार धोना

□ ऑफ़िशियल सोर्सेस से जानकारी को प्राथमिकता देते हैं

□ इनमें से कोई भी नहीं / निश्चित नहीं

Q23. अगर आगे चलकर एक सुरक्षित और असरदार निपाह वैक्सीन उपलब्ध हो जाए, तो क्या आप टीका लगवाने के लिए तैयार होंगे? *(एक विकल्प) **

○ बहुत इच्छुक

○ इच्छुक

○ ठीक से पता नहीं है

○ अनिच्छुक

○ बहुत अनिच्छुक

Q24. अगर आगे चलकर निपाह के लिए एक सुरक्षित और असरदार उपचार उपलब्ध हो जाए, तो क्या आप उसका इस्तेमाल करने के लिए तैयार होंगे? *(एक विकल्प) **

○ बहुत इच्छुक

○ इच्छुक

○ ठीक से पता नहीं है

○ अनिच्छुक

○ बहुत अनिच्छुक

प्रश्नावली समाप्त हुई। भाग लेने के लिए आपका धन्यवाद!

Supplementary Methods: Data Processing and Variable Definitions

Data Pre-processing and Handling of Sparse Categories

Prior to the mediation and predictive modeling analyses, rigorous data cleaning and dimensionality reduction were conducted to ensure computational stability and prevent over-parameterization. A critical pre-processing step involved the management of extremely sparse covariate categories, which can introduce singular matrices during regression estimations and destabilize the 10-fold cross-validation process within the predictive modeling algorithms. Consequently, participants identifying as "Third gender / transgender / prefer not to disclose" and those selecting "Other / Not sure" as their primary trusted information source were excluded from the analytical dataset due to excessive sparsity.

To establish robust epidemiological baselines for the regression models, reference categories for all categorical covariates were strictly constrained to the lowest risk or fundamental socio-demographic states (e.g., male gender, rural residence, absence of vulnerable family members, $\leqslant 5$ daily close contacts, and official health departments as the default trusted information source). Following these variable transformations and exclusions, complete case analysis (listwise deletion) was employed. Only participants with complete data across the predefined covariate set, mediators, and outcome variables were retained in the final statistical modeling (final n = 368 for Group A, 371 for Group B, and 373 for the no nudge group).

Variable Transformations and Dimensionality Reduction

To preserve degrees of freedom while maintaining epidemiological relevance, several socio-demographic and behavioral covariates underwent targeted dimensionality reduction. For instance, sparse occupational categories were systematically collapsed into broader analytical groups, merging healthcare, veterinary, and livestock workers into a single high-risk occupational tier. Continuous variables and ordinal scales were standardized or reverse-coded where necessary to ensure that higher numerical values consistently represented elevated risks, increased psychological constructs, or positive protective behaviors.

Specifically, for the calculation of the total Health Belief Model (HBM) score, the perceived barriers dimension was reverse-coded using a mathematical transformation of 6 minus the raw score to align the directional valence. Regarding the three primary protective behaviors, promptly seeking medical care was operationalized as a binary outcome appropriate for logistic regression link functions, whereas vaccination intention and treatment intention were treated as continuous variables on a 5-point scale for linear regression modeling. The specific recoding logic, aggregation strategies for macro-level exposures used in the sensitivity analyses, and the operational definitions for all analytical variables are detailed in the following table.

Data Processing and Variable Definitions Table

Analytical Variable	Original Survey Item	Coding Strategy and Definition
**Protective Behaviors**		
Promptly seeking medical care	Q22 option 2 (Multiple choice checkbox)	Converted from a checklist selection to a binary variable for logistic modeling (1 = Checked, 0 = Unchecked)
Vaccination intention	Q23 (5-point ordinal scale from 1=Very willing to 5=Very unwilling)	Reverse-coded into a continuous scale for linear modeling (1 = Very unwilling to 5 = Very willing)
Treatment intention	Q24 (5-point ordinal scale from 1=Very willing to 5=Very unwilling)	Reverse-coded into a continuous scale for linear modeling (1 = Very unwilling to 5 = Very willing)
**HBM Dimensions**		
Individual HBM dimensions	Q16 and Q21 (6 items each on a 5-point Likert scale)	Analyzed as continuous variables retaining the original 1 to 5 scale
Total HBM Score	Aggregation of the 6 items	Composite continuous score ranging from 6 to 30 with the perceived barriers dimension reverse-coded
**Cognitive & Exposure Predictors**		
Objective Knowledge Score	Q13 (6 items with True/False/Not sure options)	Composite continuous score ranging from 0 to 6 generated by summing

		correct responses
Prior Information Exposure	Q10 (4-category ordinal scale)	Collapsed into a binary proxy (Yes once or multiple times vs No or Not sure)
**Demographic & Epidemiological Covariates**		
Residence in regions with prior Nipah virus (NiV) outbreaks	Q2 (36-category nominal scale of Indian states)	Collapsed into a binary variable (Kerala or West Bengal vs all other states)
Vulnerable Family Members	Q8 (5-option multiple choice checklist)	Collapsed into a binary variable (Checked any vulnerable group vs Checked none of the above)
Occupation	Q7 (7-category nominal scale)	Collapsed into five core analytical tiers (Medical and Animal Workers, Student, Company Employee, Unemployed, and Other)
Daily Close Contacts	Q20 (7-category ordinal scale)	Collapsed into three analytical tiers (Low ≤ 5 per day, High > 5 per day, and Not sure)
Dietary Risk Exposures	Q18 and Q19 (5-point frequency scale from Almost every day to Rarely/never)	Collapsed into a binary frequency variable (At least 1 to 3 times a month vs Rarely or Never)
**Macro-level Sensitivities**		
Macro-level Risk Experiences	Q14 (4-option multiple choice checklist)	Aggregated binary proxy (Checked any high-risk activity vs Checked none of the above)
Macro-level Venue Exposures	Q15 (4-option multiple choice checklist)	Aggregated binary proxy (Checked any high-risk venue vs Checked none of the above)

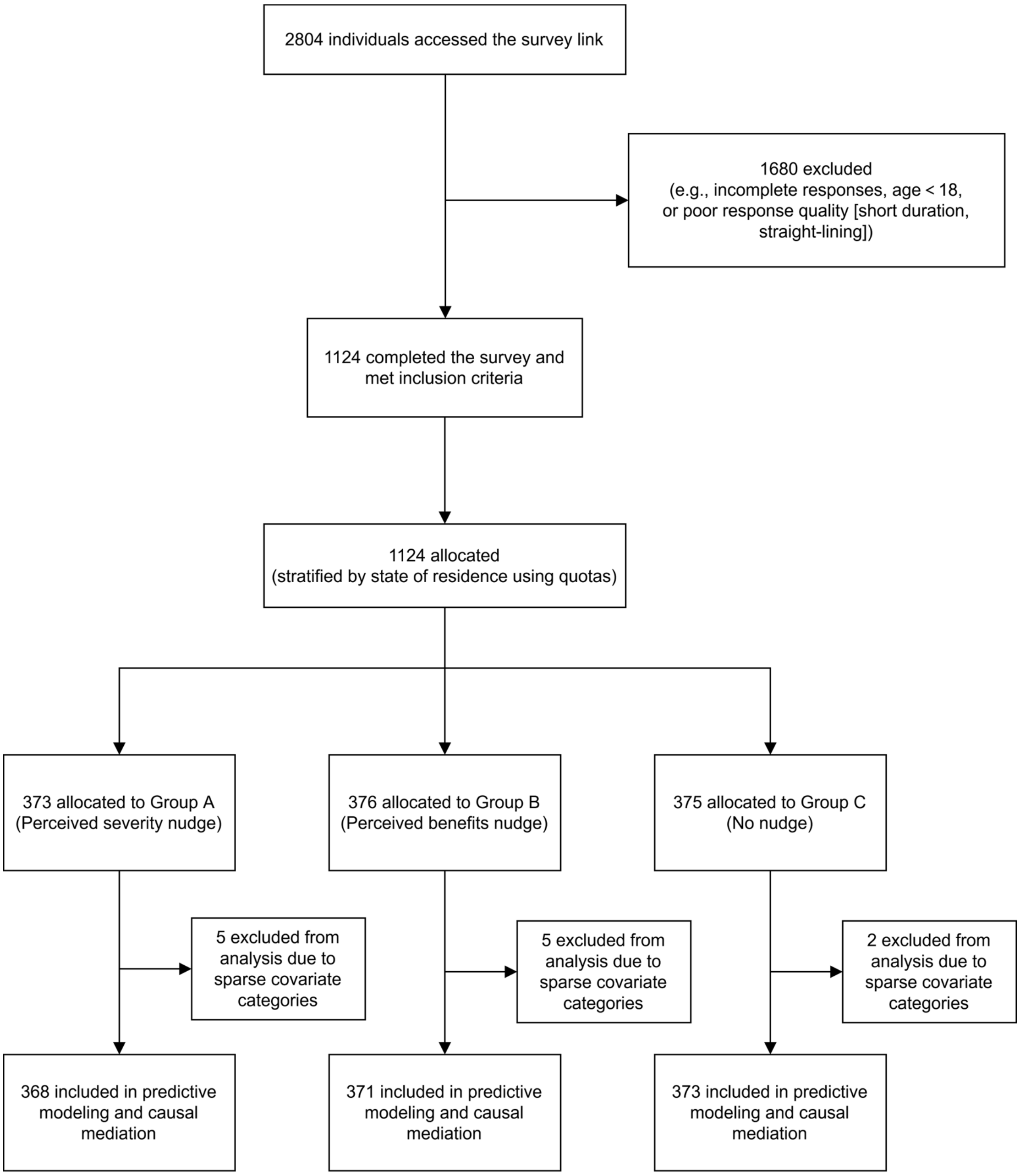

Figure S1 Study profile.

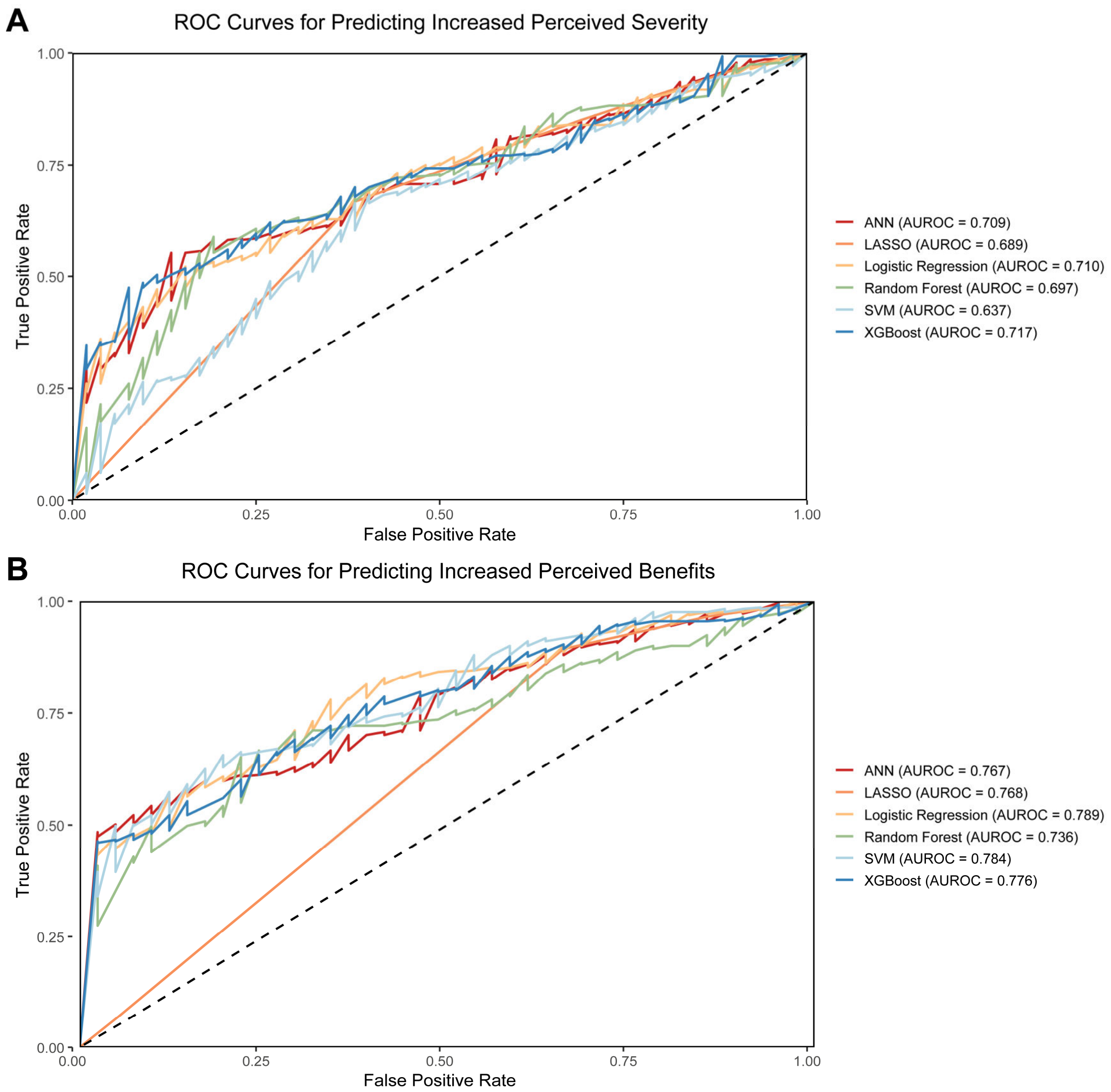

Figure S2 ROC curves of six predictive models for identifying natural susceptibility to cognitive changes.

Notes: ANN, Artificial Neural Network; AUROC, Area Under the Receiver Operating Characteristic Curve; LASSO, Least Absolute Shrinkage and Selection Operator; RF, Random Forest; ROC, Receiver Operating Characteristic; SVM, Support Vector Machine; XGBoost, eXtreme Gradient Boosting.

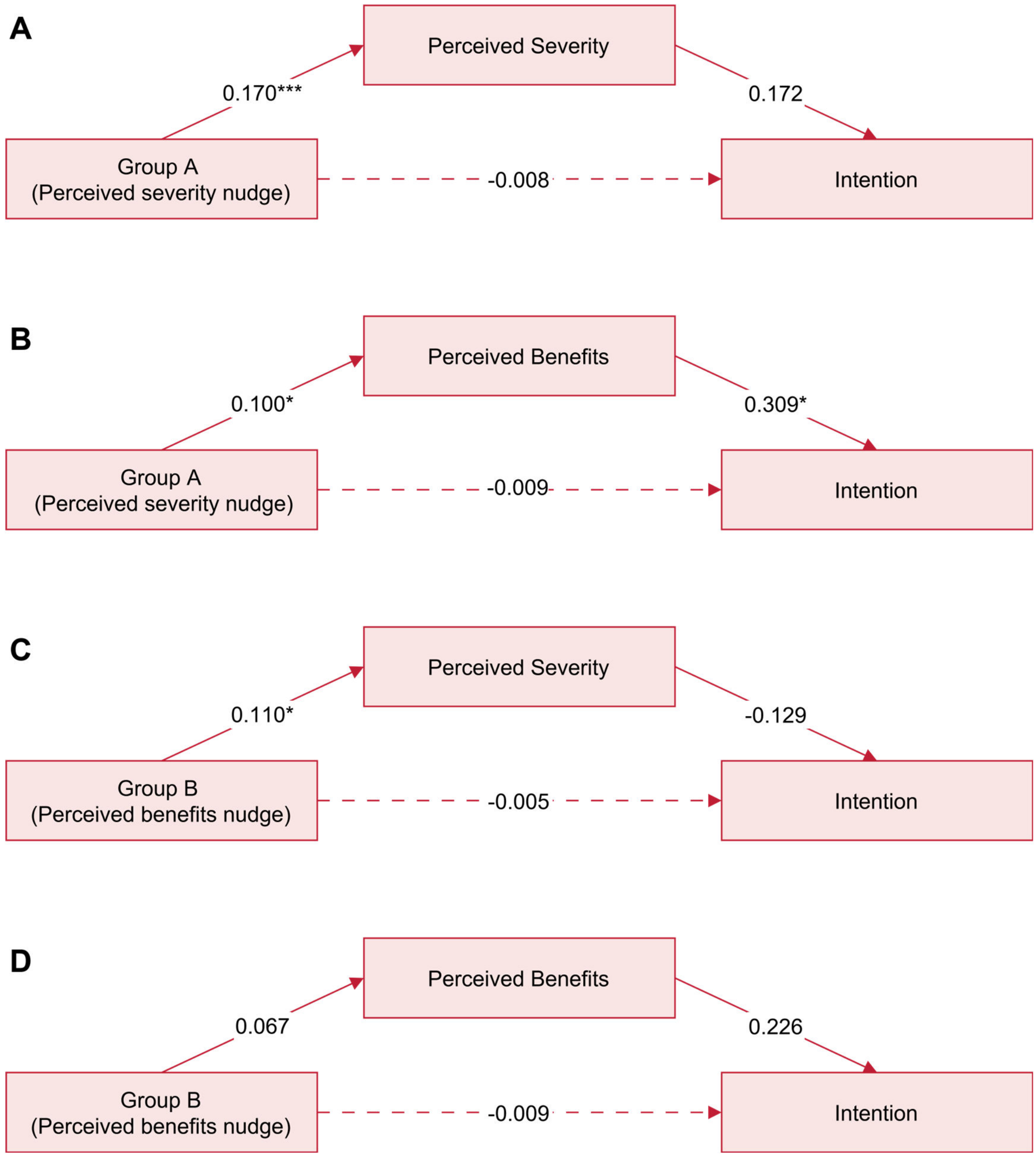

Figure S3 Mediation pathways of information nudges on promptly seeking medical care.

Notes: Models were adjusted for the corresponding baseline HBM dimension score (perceived severity or perceived benefits), frequency of handling raw pork, frequency of drinking raw date palm sap, daily close contacts, most trusted information source, objective knowledge score about NiV, residence in regions with prior NiV outbreaks, gender, age, education level, and presence of vulnerable family members. Values on the arrows represent path coefficients (β). Solid arrows indicate the indirect pathways mapping the effect of the information nudge on the mediator (path a) and the mediator on the outcome (path b). The dashed arrow indicates the direct effect of the information nudge on the outcome. *** p<0.001, ** p<0.01, * p<0.05. HBM, Health Belief Model; NiV, Nipah virus.

Table S1 Predictive performance of machine learning models and logistic regression for identifying susceptibility to cognitive changes.

Outcome / Model	AUROC (95% CI)	Sensitivity	Specificity
**Increased Perceived Severity (Δ Severity > 0)**			
**XGBoost**	**0.717 (0.654, 0.779)**	**0.475**	**0.923**
Logistic Regression	0.710 (0.645, 0.775)	0.521	0.846
ANN	0.709 (0.644, 0.774)	0.554	0.865
Random Forest	0.697 (0.624, 0.769)	0.589	0.808
LASSO	0.689 (0.623, 0.755)	0.668	0.615
SVM	0.637 (0.557, 0.718)	0.664	0.615
**Increased Perceived Benefits (Δ Benefits > 0)**			
**Logistic Regression**	**0.789 (0.727, 0.850)**	**0.564**	**0.878**
SVM	0.784 (0.721, 0.847)	0.656	0.805
XGBoost	0.776 (0.713, 0.838)	0.460	1.000
LASSO	0.768 (0.717, 0.818)	0.450	1.000
ANN	0.767 (0.706, 0.829)	0.474	1.000
Random Forest	0.736 (0.670, 0.802)	0.653	0.780

Notes: Models were adjusted for the corresponding baseline HBM dimension score (perceived severity or perceived benefits), frequency of handling raw pork, frequency of drinking raw date palm sap, daily close contacts, most trusted information source, objective knowledge score about NiV, residence in regions with prior NiV outbreaks, gender, age, education level, and presence of vulnerable family members. ANN, Artificial Neural Network; AUROC, Area Under the Receiver Operating Characteristic Curve; CI, Confidence Interval; HBM, Health Belief Model; LASSO, Least Absolute Shrinkage and Selection Operator; NiV, Nipah virus; SVM, Support Vector Machine; XGBoost, eXtreme Gradient Boosting.

Table S2 Mediation effects of severity- and benefit-focused information nudges on vaccination and treatment intentions.

Panel A: Pathways predicting promptly seeking medical care.

**Information Nudge (X)**	**Mediator (M)**	**Path a: X → M β (95% CI)**	**Path b: M → Y β (95% CI)**	**Direct Effect β (95% CI)**	**Indirect Effect β (95% CI)**	**Total Effect β (95% CI)**
**Group A (Perceived severity nudge) vs. No nudge**	Perceived Susceptibility	0.062 (-0.045, 0.170)	-0.126 (-0.350, 0.098)	-0.001 (-0.063, 0.061)	-0.001 (-0.008, 0.002)	-0.003 (-0.063, 0.060)
	Perceived Severity	0.170 (0.071, 0.269)***	0.172 (-0.071, 0.417)	-0.008 (-0.070, 0.054)	0.005 (-0.001, 0.016)	-0.002 (-0.062, 0.063)
	Perceived Benefits	0.100 (0.013, 0.188)*	0.309 (0.044, 0.581)*	-0.009 (-0.069, 0.054)	0.006 (0.000, 0.014)	-0.003 (-0.062, 0.060)
	Perceived Barriers	-0.059 (-0.168, 0.049)	-0.110 (-0.333, 0.112)	-0.005 (-0.065, 0.058)	0.001 (-0.002, 0.005)	-0.004 (-0.064, 0.058)
	Cues to Action	0.203 (0.110, 0.296)***	0.050 (-0.204, 0.304)	-0.006 (-0.065, 0.059)	0.002 (-0.008, 0.012)	-0.004 (-0.063, 0.060)
	Self-Efficacy	0.079 (-0.004, 0.163)	0.081 (-0.194, 0.356)	-0.007 (-0.068, 0.056)	0.001 (-0.004, 0.006)	-0.006 (-0.068, 0.055)
**Group B (Perceived benefits nudge) vs. No nudge**	Perceived Susceptibility	0.058 (-0.041, 0.157)	-0.039 (-0.278, 0.200)	-0.005 (-0.064, 0.057)	0.000 (-0.004, 0.003)	-0.006 (-0.065, 0.057)
	Perceived Severity	0.110 (0.011, 0.208)*	-0.129 (-0.372, 0.111)	-0.005 (-0.065, 0.055)	-0.003 (-0.010, 0.003)	-0.008 (-0.068, 0.052)
	Perceived Benefits	0.067 (-0.010, 0.144)	0.226 (-0.079, 0.537)	-0.009 (-0.067, 0.051)	0.003 (-0.001, 0.010)	-0.006 (-0.066, 0.054)
	Perceived Barriers	-0.058 (-0.151, 0.035)	0.189 (-0.072, 0.456)	-0.004 (-0.061, 0.059)	-0.002 (-0.009, 0.002)	-0.006 (-0.064, 0.054)

	Cues to Action	0.289 (0.204, 0.375)***	0.068 (-0.209, 0.349)	-0.011 (-0.072, 0.053)	0.004 (-0.012, 0.020)	-0.007 (-0.067, 0.055)
	Self-Efficacy	0.071 (0.000, 0.143)	0.198 (-0.124, 0.526)	-0.011 (-0.072, 0.048)	0.003 (-0.002, 0.012)	-0.008 (-0.068, 0.052)

Panel B: Pathways predicting vaccination intention.

Information Nudge (X)	Mediator (M)	Path a: X → M<br>β (95% CI)	Path b: M → Y<br>β (95% CI)	Direct Effect β (95% CI)	Indirect Effect<br>β (95% CI)	Total Effect<br>β (95% CI)
**Group A (Perceived severity nudge) vs. No nudge**	Perceived Susceptibility	0.062 (-0.045, 0.170)	-0.005 (-0.082, 0.071)	0.011 (-0.104, 0.127)	0.000 (-0.011, 0.007)	0.011 (-0.104, 0.129)
	Perceived Severity	0.170 (0.071, 0.269)***	0.085 (0.002, 0.167)*	-0.007 (-0.114, 0.110)	0.014 (-0.001, 0.032)	0.008 (-0.094, 0.125)
	Perceived Benefits	0.100 (0.013, 0.188)*	0.058 (-0.036, 0.152)	0.007 (-0.101, 0.127)	0.006 (-0.005, 0.020)	0.013 (-0.094, 0.131)
	Perceived Barriers	-0.059 (-0.168, 0.049)	-0.017 (-0.093, 0.060)	0.014 (-0.096, 0.133)	0.001 (-0.006, 0.008)	0.015 (-0.095, 0.132)
	Cues to Action	0.203 (0.110, 0.296)***	0.039 (-0.048, 0.127)	0.000 (-0.096, 0.119)	0.008 (-0.016, 0.034)	0.008 (-0.097, 0.128)
	Self-Efficacy	0.079 (-0.004, 0.163)	0.098 (-0.001, 0.196)	-0.004 (-0.116, 0.109)	0.008 (-0.001, 0.023)	0.004 (-0.101, 0.119)
**Group B (Perceived benefits nudge) vs. No nudge**	Perceived Susceptibility	0.058 (-0.041, 0.157)	0.028 (-0.051, 0.106)	0.077 (-0.026, 0.181)	0.002 (-0.002, 0.010)	0.078 (-0.024, 0.183)
	Perceived Severity	0.110 (0.011, 0.208)*	0.043 (-0.036, 0.122)	0.068 (-0.032, 0.173)	0.005 (-0.004, 0.017)	0.073 (-0.033, 0.171)
	Perceived Benefits	0.067 (-0.010,	0.122 (0.022,	0.070 (-0.035,	0.008 (-0.002,	0.078 (-0.025,

		0.144)	0.222)*	0.171)	0.025)	0.176)
	Perceived Barriers	-0.058 (-0.151, 0.035)	0.012 (-0.072, 0.095)	0.080 (-0.025, 0.188)	-0.001 (-0.008, 0.007)	0.079 (-0.026, 0.185)
	Cues to Action	0.289 (0.204, 0.375)***	0.082 (-0.007, 0.171)	0.042 (-0.063, 0.145)	0.024 (0.001, 0.054)*	0.066 (-0.038, 0.169)
	Self-Efficacy	0.071 (0.000, 0.143)	0.074 (-0.033, 0.182)	0.070 (-0.031, 0.167)	0.005 (-0.002, 0.021)	0.075 (-0.026, 0.172)

Panel C: Pathways predicting treatment intention.

**Information Nudge (X)**	**Mediator (M)**	**Path a: X → M**<br>**β (95% CI)**	**Path b: M → Y**<br>**β (95% CI)**	**Direct Effect β (95% CI)**	**Indirect Effect**<br>**β (95% CI)**	**Total Effect**<br>**β (95% CI)**
**Group A (Perceived severity nudge) vs. No nudge**	Perceived Susceptibility	0.062 (-0.045, 0.170)	0.032 (-0.038, 0.102)	0.059 (-0.042, 0.158)	0.002 (-0.003, 0.010)	0.061 (-0.036, 0.160)
	Perceived Severity	0.170 (0.071, 0.269)***	0.092 (0.017, 0.168)*	0.043 (-0.051, 0.141)	0.016 (0.002, 0.032)*	0.059 (-0.036, 0.158)
	Perceived Benefits	0.100 (0.013, 0.188)*	0.085 (0.000, 0.171)	0.054 (-0.040, 0.155)	0.009 (-0.003, 0.022)	0.062 (-0.032, 0.163)
	Perceived Barriers	-0.059 (-0.168, 0.049)	0.002 (-0.068, 0.071)	0.064 (-0.035, 0.162)	0.000 (-0.007, 0.006)	0.064 (-0.035, 0.164)
	Cues to Action	0.203 (0.110, 0.296)***	0.035 (-0.046, 0.115)	0.052 (-0.045, 0.152)	0.007 (-0.015, 0.028)	0.059 (-0.042, 0.159)
	Self-Efficacy	0.079 (-0.004, 0.163)	0.148 (0.060, 0.237)**	0.040 (-0.051, 0.141)	0.012 (-0.001, 0.027)	0.052 (-0.044, 0.149)
**Group B (Perceived benefits nudge) vs. No nudge**	Perceived Susceptibility	0.058 (-0.041, 0.157)	-0.005 (-0.079, 0.068)	0.054 (-0.042, 0.153)	0.000 (-0.005, 0.006)	0.054 (-0.040, 0.152)
	Perceived Severity	0.110 (0.011, 0.208)*	0.033 (-0.041, 0.107)	0.044 (-0.052, 0.145)	0.004 (-0.005, 0.015)	0.048 (-0.045, 0.148)

	Perceived Benefits	0.067 (-0.010, 0.144)	0.099 (0.004, 0.193)*	0.046 (-0.046, 0.150)	0.007 (-0.003, 0.022)	0.052 (-0.041, 0.155)
	Perceived Barriers	-0.058 (-0.151, 0.035)	0.025 (-0.053, 0.104)	0.057 (-0.039, 0.154)	-0.001 (-0.010, 0.005)	0.056 (-0.040, 0.153)
	Cues to Action	0.289 (0.204, 0.375)***	0.096 (0.012, 0.180)*	0.016 (-0.077, 0.118)	0.028 (0.002, 0.055)*	0.044 (-0.049, 0.142)
	Self-Efficacy	0.071 (0.000, 0.143)	0.108 (0.008, 0.208)*	0.041 (-0.054, 0.145)	0.008 (-0.001, 0.025)	0.048 (-0.045, 0.149)

Notes: Models were adjusted for the corresponding baseline HBM dimension score, frequency of handling raw pork, frequency of drinking raw date palm sap, daily close contacts, most trusted information source, objective knowledge score about NiV, residence in regions with prior NiV outbreaks, gender, age, education level, and presence of vulnerable family members. Path a represents the effect of the information nudge on the mediator. Path b represents the effect of the mediator on the outcome, controlling for the information nudge. *** $p<0.001$, ** $p<0.01$, * $p<0.05$. CI, confidence interval; HBM, Health Belief Model; NiV, Nipah virus.

Table S3 Sensitivity analysis of predictive models using a minimal feature set.

Outcome / Model	AUROC (95% CI)	Sensitivity	Specificity
**Increased Perceived Severity (Δ Severity > 0)**			
**Logistic Regression**	**0.760 (0.699, 0.822)**	**0.625**	**0.830**
ANN	0.755 (0.693, 0.818)	0.661	0.774
LASSO	0.751 (0.690, 0.813)	0.664	0.755
XGBoost	0.724 (0.659, 0.790)	0.686	0.717
Random Forest	0.676 (0.610, 0.742)	0.399	0.906
SVM	0.600 (0.510, 0.690)	0.760	0.472
**Increased Perceived Benefits (Δ Benefits > 0)**			
**ANN**	**0.832 (0.778, 0.885)**	**0.735**	**0.786**
LASSO	0.830 (0.776, 0.884)	0.731	0.786
Logistic Regression	0.829 (0.774, 0.883)	0.724	0.786
XGBoost	0.814 (0.758, 0.869)	0.599	0.929
SVM	0.772 (0.703, 0.841)	0.786	0.667
Random Forest	0.770 (0.705, 0.836)	0.599	0.905

Notes: Models were adjusted for the corresponding baseline HBM dimension score (perceived severity or perceived benefits), residence in regions with prior NiV outbreaks, gender, age, education level, and presence of vulnerable family members. ANN, Artificial Neural Network; AUROC, Area Under the Receiver Operating Characteristic Curve; CI, Confidence Interval; HBM, Health Belief Model; LASSO, Least Absolute Shrinkage and Selection Operator; NiV, Nipah virus; SVM, Support Vector Machine; XGBoost, eXtreme Gradient Boosting.

Table S4 Sensitivity analysis of mediation models using a minimal covariate adjustment set.

Panel A: Pathways predicting promptly seeking medical care.

Information Nudge (X)	Mediator (M)	Path a: X → M β (95% CI)	Path b: M → Y β (95% CI)	Direct Effect β (95% CI)	Indirect Effect β (95% CI)	Total Effect β (95% CI)
**Group A (Perceived severity nudge) vs. No nudge**	Perceived Severity	0.167 (0.069, 0.265)***	0.195 (-0.043, 0.437)	-0.001 (-0.071, 0.065)	0.006 (0.000, 0.017)	0.006 (-0.062, 0.071)
	Perceived Benefits	0.092 (0.005, 0.180)*	0.342 (0.084, 0.606)**	-0.002 (-0.072, 0.064)	0.006 (-0.001, 0.013)	0.004 (-0.064, 0.071)
	Cues to action	0.201 (0.109, 0.294)***	0.126 (-0.124, 0.379)	0.000 (-0.071, 0.067)	0.005 (-0.005, 0.017)	0.005 (-0.063, 0.072)
**Group B (Perceived benefits nudge) vs. No nudge**	Perceived Severity	0.103 (0.006, 0.200)*	-0.100 (-0.340, 0.134)	-0.008 (-0.076, 0.058)	-0.002 (-0.009, 0.003)	-0.010 (-0.077, 0.055)
	Perceived Benefits	0.059 (-0.018, 0.137)	0.290 (-0.003, 0.592)	-0.012 (-0.080, 0.052)	0.003 (-0.001, 0.011)	-0.009 (-0.074, 0.056)
	Cues to action	0.288 (0.202, 0.374)***	0.159 (-0.108, 0.432)	-0.019 (-0.091, 0.053)	0.009 (-0.008, 0.025)	-0.010 (-0.075, 0.056)

Panel B: Pathways predicting vaccination intention.

Information Nudge (X)	Mediator (M)	Path a: X → M β (95% CI)	Path b: M → Y β (95% CI)	Direct Effect β (95% CI)	Indirect Effect β (95% CI)	Total Effect β (95% CI)
**Group A (Perceived severity nudge) vs. No nudge**	Perceived Severity	0.167 (0.069, 0.265)***	0.100 (0.015, 0.184)*	-0.025 (-0.138, 0.090)	0.017 (0.001, 0.036)*	-0.008 (-0.124, 0.105)
	Perceived Benefits	0.092 (0.005, 0.180)*	0.059 (-0.036, 0.153)	-0.008 (-0.123, 0.104)	0.005 (-0.004, 0.018)	-0.003 (-0.122, 0.109)

	Cues to Action	0.201 (0.109, 0.294)***	0.052 (-0.037, 0.140)	-0.017 (-0.138, 0.089)	0.010 (-0.013, 0.035)	-0.006 (-0.129, 0.102)
**Group B (Perceived benefits nudge) vs. No nudge**	Perceived Severity	0.103 (0.006, 0.200)*	0.042 (-0.039, 0.123)	0.036 (-0.072, 0.135)	0.004 (-0.005, 0.020)	0.040 (-0.067, 0.144)
	Perceived Benefits	0.059 (-0.018, 0.137)	0.140 (0.038, 0.241)**	0.038 (-0.074, 0.148)	0.008 (-0.002, 0.023)	0.046 (-0.063, 0.155)
	Cues to Action	0.288 (0.202, 0.374)***	0.097 (0.007, 0.187)*	0.008 (-0.100, 0.115)	0.028 (-0.001, 0.062)	0.036 (-0.069, 0.139)

Panel C: Pathways predicting treatment intention.

**Information Nudge (X)**	**Mediator (M)**	**Path a: X → M**<br>**β (95% CI)**	**Path b: M → Y**<br>**β (95% CI)**	**Direct Effect β (95% CI)**	**Indirect Effect**<br>**β (95% CI)**	**Total Effect**<br>**β (95% CI)**
**Group A (Perceived severity nudge) vs. No nudge**	Perceived Severity	0.167 (0.069, 0.265)***	0.101 (0.025, 0.177)**	0.030 (-0.074, 0.129)	0.017 (0.003, 0.033)**	0.047 (-0.058, 0.148)
	Perceived Benefits	0.092 (0.005, 0.180)*	0.090 (0.005, 0.176)*	0.043 (-0.059, 0.140)	0.008 (-0.002, 0.022)	0.051 (-0.055, 0.151)
	Cues to Action	0.201 (0.109, 0.294)***	0.053 (-0.027, 0.134)	0.038 (-0.065, 0.141)	0.011 (-0.010, 0.033)	0.049 (-0.059, 0.149)
**Group B (Perceived benefits nudge) vs. No nudge**	Perceived Severity	0.103 (0.006, 0.200)*	0.030 (-0.046, 0.106)	0.012 (-0.088, 0.106)	0.003 (-0.007, 0.016)	0.015 (-0.083, 0.111)
	Perceived Benefits	0.059 (-0.018, 0.137)	0.136 (0.042, 0.231)**	0.011 (-0.088, 0.104)	0.008 (-0.002, 0.024)	0.019 (-0.082, 0.114)
	Cues to Action	0.288 (0.202, 0.374)***	0.117 (0.032, 0.202)**	-0.021 (-0.125, 0.075)	0.034 (0.009, 0.063)**	0.012 (-0.085, 0.105)

Notes: Models were adjusted for the corresponding baseline HBM dimension score (perceived severity, perceived benefits, or cues to action), residence in regions with prior NiV outbreaks, gender, age, education level, and presence of vulnerable family members. Path a represents the effect of the

information nudge on the mediator. Path b represents the effect of the mediator on the outcome, controlling for the information nudge. *** $p<0.001$, ** $p<0.01$, * $p<0.05$. CI, confidence interval; HBM, Health Belief Model; NiV, Nipah virus.

Table S5 Sensitivity analysis of mediation models using subjective cognitive proxies.

Panel A: Pathways predicting promptly seeking medical care.

Information Nudge (X)	Mediator (M)	Path a: X → M β (95% CI)	Path b: M → Y β (95% CI)	Direct Effect β (95% CI)	Indirect Effect β (95% CI)	Total Effect β (95% CI)
**Group A (Perceived severity nudge) vs. No nudge**	Perceived Severity	0.168 (0.069, 0.268)***	0.176 (-0.068, 0.423)	-0.010 (-0.070, 0.056)	0.006 (-0.002, 0.017)	-0.004 (-0.064, 0.062)
	Perceived Benefits	0.097 (0.010, 0.185)*	0.298 (0.030, 0.572)*	-0.010 (-0.070, 0.055)	0.005 (-0.001, 0.013)	-0.005 (-0.063, 0.059)
	Cues to Action	0.200 (0.107, 0.293)***	0.036 (-0.219, 0.292)	-0.006 (-0.064, 0.058)	0.001 (-0.008, 0.011)	-0.005 (-0.064, 0.061)
**Group B (Perceived benefits nudge) vs. No nudge**	Perceived Severity	0.104 (0.005, 0.202)*	-0.090 (-0.333, 0.150)	-0.015 (-0.074, 0.048)	-0.002 (-0.008, 0.004)	-0.017 (-0.075, 0.048)
	Perceived Benefits	0.061 (-0.015, 0.138)	0.189 (-0.118, 0.501)	-0.018 (-0.075, 0.045)	0.002 (-0.001, 0.009)	-0.015 (-0.075, 0.047)
	Cues to Action	0.284 (0.199, 0.370)***	0.061 (-0.216, 0.344)	-0.019 (-0.081, 0.047)	0.003 (-0.012, 0.019)	-0.016 (-0.074, 0.050)

Panel B: Pathways predicting vaccination intention.

Information Nudge (X)	Mediator (M)	Path a: X → M β (95% CI)	Path b: M → Y β (95% CI)	Direct Effect β (95% CI)	Indirect Effect β (95% CI)	Total Effect β (95% CI)
**Group A (Perceived severity nudge) vs. No nudge**	Perceived Severity	0.168 (0.069, 0.268)***	0.086 (0.004, 0.169)*	-0.009 (-0.115, 0.111)	0.015 (-0.002, 0.032)	0.006 (-0.102, 0.123)
	Perceived Benefits	0.097 (0.010, 0.185)*	0.057 (-0.037, 0.152)	0.006 (-0.104, 0.127)	0.006 (-0.004, 0.018)	0.011 (-0.101, 0.128)

	Cues to Action	0.200 (0.107, 0.293)***	0.042 (-0.046, 0.130)	-0.002 (-0.102, 0.116)	0.008 (-0.015, 0.035)	0.007 (-0.098, 0.127)
**Group B (Perceived benefits nudge) vs. No nudge**	Perceived Severity	0.104 (0.005, 0.202)*	0.048 (-0.031, 0.127)	0.059 (-0.042, 0.163)	0.005 (-0.003, 0.017)	0.064 (-0.040, 0.163)
	Perceived Benefits	0.061 (-0.015, 0.138)	0.118 (0.017, 0.218)*	0.063 (-0.039, 0.167)	0.007 (-0.003, 0.023)	0.070 (-0.033, 0.167)
	Cues to Action	0.284 (0.199, 0.370)***	0.082 (-0.007, 0.172)	0.037 (-0.070, 0.137)	0.023 (0.001, 0.054)*	0.060 (-0.045, 0.160)

Panel C: Pathways predicting treatment intention.

**Information Nudge (X)**	**Mediator (M)**	**Path a: X → M**<br>**β (95% CI)**	**Path b: M → Y**<br>**β (95% CI)**	**Direct Effect β (95% CI)**	**Indirect Effect**<br>**β (95% CI)**	**Total Effect**<br>**β (95% CI)**
**Group A (Perceived severity nudge) vs. No nudge**	Perceived Severity	0.168 (0.069, 0.268)***	0.093 (0.018, 0.168)*	0.040 (-0.058, 0.142)	0.016 (0.002, 0.031)**	0.056 (-0.043, 0.155)
	Perceived Benefits	0.097 (0.010, 0.185)*	0.077 (-0.008, 0.163)	0.052 (-0.046, 0.152)	0.008 (-0.004, 0.022)	0.059 (-0.040, 0.158)
	Cues to Action	0.200 (0.107, 0.293)***	0.030 (-0.050, 0.110)	0.051 (-0.047, 0.150)	0.006 (-0.017, 0.026)	0.057 (-0.042, 0.152)
**Group B (Perceived benefits nudge) vs. No nudge**	Perceived Severity	0.104 (0.005, 0.202)*	0.045 (-0.028, 0.119)	0.037 (-0.054, 0.136)	0.005 (-0.003, 0.018)	0.042 (-0.049, 0.141)
	Perceived Benefits	0.061 (-0.015, 0.138)	0.095 (0.001, 0.189)*	0.040 (-0.048, 0.147)	0.006 (-0.004, 0.021)	0.046 (-0.045, 0.150)
	Cues to Action	0.284 (0.199, 0.370)***	0.096 (0.012, 0.180)*	0.012 (-0.079, 0.110)	0.027 (0.001, 0.055)*	0.039 (-0.053, 0.139)

Notes: Models were adjusted for the corresponding baseline HBM dimension score (perceived severity, perceived benefits or cues to action), frequency of handling raw pork, frequency of drinking raw date palm sap, average close contacts per day, most trusted information source, prior awareness of NiV

(heard of NiV), NiV information exposure in the past 12 months, residence in regions with prior NiV outbreaks, gender, age, highest education, and presence of vulnerable family members. Path a represents the effect of the information nudge on the mediator. Path b represents the effect of the mediator on the outcome (vaccination or treatment intention), controlling for the information nudge. *** $p<0.001$, ** $p<0.01$, * $p<0.05$. CI, confidence interval; HBM, Health Belief Model; NiV, Nipah virus.

Table S6 Sensitivity analysis of mediation models using generalised high-risk exposure histories.

Panel A: Pathways predicting promptly seeking medical care.

Information Nudge (X)	Mediator (M)	Path a: X → M β (95% CI)	Path b: M → Y β (95% CI)	Direct Effect β (95% CI)	Indirect Effect β (95% CI)	Total Effect β (95% CI)
**Group A (Perceived severity nudge) vs. No nudge**	Perceived Severity	0.173 (0.074, 0.271)***	0.187 (-0.059, 0.435)	-0.002 (-0.065, 0.064)	0.006 (-0.001, 0.017)	0.004 (-0.058, 0.070)
	Perceived Benefits	0.098 (0.011, 0.186)*	0.335 (0.066, 0.608)*	-0.003 (-0.065, 0.065)	0.006 (0.000, 0.014)	0.003 (-0.061, 0.067)
	Cues to Action	0.206 (0.113, 0.298)***	0.032 (-0.227, 0.291)	0.002 (-0.061, 0.069)	0.001 (-0.008, 0.012)	0.004 (-0.059, 0.069)
**Group B (Perceived benefits nudge) vs. No nudge**	Perceived Severity	0.104 (0.006, 0.202)*	-0.114 (-0.356, 0.124)	-0.001 (-0.060, 0.062)	-0.002 (-0.009, 0.003)	-0.003 (-0.060, 0.060)
	Perceived Benefits	0.065 (-0.013, 0.143)	0.283 (-0.017, 0.590)	-0.005 (-0.062, 0.056)	0.003 (-0.001, 0.011)	-0.002 (-0.061, 0.060)
	Cues to Action	0.296 (0.209, 0.382)***	0.116 (-0.158, 0.394)	-0.009 (-0.070, 0.055)	0.006 (-0.009, 0.024)	-0.002 (-0.060, 0.060)

Panel B: Pathways predicting vaccination intention.

Information Nudge (X)	Mediator (M)	Path a: X → M β (95% CI)	Path b: M → Y β (95% CI)	Direct Effect β (95% CI)	Indirect Effect β (95% CI)	Total Effect β (95% CI)
**Group A (Perceived severity nudge) vs. No nudge**	Perceived Severity	0.173 (0.074, 0.271)***	0.084 (0.001, 0.166)*	0.002 (-0.108, 0.118)	0.014 (-0.001, 0.032)	0.016 (-0.089, 0.130)
	Perceived Benefits	0.098 (0.011, 0.186)*	0.053 (-0.041, 0.146)	0.017 (-0.089, 0.139)	0.005 (-0.005, 0.019)	0.022 (-0.085, 0.138)

	Cues to Action	0.206 (0.113, 0.298)***	0.036 (-0.052, 0.123)	0.008 (-0.088, 0.124)	0.007 (-0.016, 0.034)	0.015 (-0.089, 0.137)
**Group B (Perceived benefits nudge) vs. No nudge**	Perceived Severity	0.104 (0.006, 0.202)*	0.041 (-0.037, 0.120)	0.076 (-0.023, 0.184)	0.004 (-0.004, 0.017)	0.080 (-0.020, 0.186)
	Perceived Benefits	0.065 (-0.013, 0.143)	0.125 (0.026, 0.223)*	0.076 (-0.023, 0.178)	0.008 (-0.002, 0.025)	0.084 (-0.017, 0.187)
	Cues to Action	0.296 (0.209, 0.382)***	0.079 (-0.009, 0.167)	0.051 (-0.049, 0.158)	0.023 (0.000, 0.055)*	0.074 (-0.025, 0.182)

Panel C: Pathways predicting treatment intention.

**Information Nudge (X)**	**Mediator (M)**	**Path a: X → M**	**Path b: M → Y**	**Direct Effect**	**Indirect Effect**	**Total Effect**
		**β (95% CI)**	**β (95% CI)**	**β (95% CI)**	**β (95% CI)**	**β (95% CI)**
**Group A (Perceived severity nudge) vs. No nudge**	Perceived Severity	0.173 (0.074, 0.271)***	0.094 (0.020, 0.169)*	0.044 (-0.053, 0.137)	0.016 (0.002, 0.034)*	0.061 (-0.038, 0.153)
	Perceived Benefits	0.098 (0.011, 0.186)*	0.083 (-0.001, 0.167)	0.056 (-0.040, 0.150)	0.008 (-0.002, 0.022)	0.065 (-0.034, 0.154)
	Cues to Action	0.206 (0.113, 0.298)***	0.028 (-0.052, 0.107)	0.054 (-0.044, 0.145)	0.006 (-0.016, 0.026)	0.060 (-0.038, 0.151)
**Group B (Perceived benefits nudge) vs. No nudge**	Perceived Severity	0.104 (0.006, 0.202)*	0.038 (-0.035, 0.112)	0.049 (-0.045, 0.153)	0.004 (-0.005, 0.016)	0.053 (-0.043, 0.154)
	Perceived Benefits	0.065 (-0.013, 0.143)	0.111 (0.019, 0.203)*	0.048 (-0.044, 0.153)	0.007 (-0.003, 0.023)	0.055 (-0.039, 0.158)
	Cues to Action	0.296 (0.209, 0.382)***	0.101 (0.018, 0.184)*	0.019 (-0.076, 0.124)	0.030 (0.006, 0.058)*	0.049 (-0.045, 0.151)

Notes: Models were adjusted for the corresponding baseline HBM dimension score (perceived severity, perceived benefits, cues to action), recent high-risk experiences (past 3 months), recent high-risk venue exposures (past 12 months), most trusted information source, baseline NiV knowledge score, residence

in regions with prior NiV outbreaks, gender, age, highest education, and presence of vulnerable family members. Path a represents the effect of the information nudge on the mediator. Path b represents the effect of the mediator on the outcome (vaccination or treatment intention), controlling for the information nudge. *** $p<0.001$, ** $p<0.01$, * $p<0.05$. CI, confidence interval; HBM, Health Belief Model; NiV, Nipah virus.

STROBE Statement—Checklist of items that should be included in reports of *cross-sectional studies*

	Item No	Recommendation	Page No
**Title and abstract**	1	(*a*) Indicate the study's design with a commonly used term in the title or the abstract	Page 1-2
		(*b*) Provide in the abstract an informative and balanced summary of what was done and what was found	Page 1-2
**Introduction**			
Background/rationale	2	Explain the scientific background and rationale for the investigation being reported	Pages 2-4
Objectives	3	State specific objectives, including any prespecified hypotheses	Page 2-4
**Methods**			
Study design	4	Present key elements of study design early in the paper	Page 5
Setting	5	Describe the setting, locations, and relevant dates, including periods of recruitment, exposure, follow-up, and data collection	Page 5
Participants	6	(*a*) Give the eligibility criteria, and the sources and methods of selection of participants	Page 5
Variables	7	Clearly define all outcomes, exposures, predictors, potential confounders, and effect modifiers. Give diagnostic criteria, if applicable	Pages 6-8
Data sources/ measurement	8*	For each variable of interest, give sources of data and details of methods of assessment (measurement). Describe comparability of assessment methods if there is more than one group	Pages 6-8
Bias	9	Describe any efforts to address potential sources of bias	Page 8
Study size	10	Explain how the study size was arrived at	Page 5
Quantitative variables	11	Explain how quantitative variables were handled in the analyses. If applicable, describe which groupings were chosen and why	Pages 6-8
Statistical methods	12	(*a*) Describe all statistical methods, including those used to control for confounding	Pages 8
		(*b*) Describe any methods used to examine subgroups and interactions	Pages 8-9
		(*c*) Explain how missing data were addressed	Page 8
		(*d*) If applicable, describe analytical methods taking account of sampling strategy	N/A
		(*e*) Describe any sensitivity analyses	Page 9
**Results**			
Participants	13*	(a) Report numbers of individuals at each stage of study—eg numbers potentially eligible, examined for	Page 10

		eligibility, confirmed eligible, included in the study, completing follow-up, and analysed	
		(b) Give reasons for non-participation at each stage	Page 10
		(c) Consider use of a flow diagram	Page 10
Descriptive data	14*	(a) Give characteristics of study participants (eg demographic, clinical, social) and information on exposures and potential confounders	Page 10-11
		(b) Indicate number of participants with missing data for each variable of interest	Page 10-11
Outcome data	15*	Report numbers of outcome events or summary measures	Page 11
Main results	16	(*a*) Give unadjusted estimates and, if applicable, confounder-adjusted estimates and their precision (eg, 95% confidence interval). Make clear which confounders were adjusted for and why they were included	Pages 11-13
		(*b*) Report category boundaries when continuous variables were categorized	Page 10-11
		(*c*) If relevant, consider translating estimates of relative risk into absolute risk for a meaningful time period	N/A
Other analyses	17	Report other analyses done—eg analyses of subgroups and interactions, and sensitivity analyses	Page 13
**Discussion**			
Key results	18	Summarise key results with reference to study objectives	Pages 13-14
Limitations	19	Discuss limitations of the study, taking into account sources of potential bias or imprecision. Discuss both direction and magnitude of any potential bias	Page 17-18
Interpretation	20	Give a cautious overall interpretation of results considering objectives, limitations, multiplicity of analyses, results from similar studies, and other relevant evidence	Pages 14-17
Generalisability	21	Discuss the generalisability (external validity) of the study results	Page 17-18
**Other information**			
Funding	22	Give the source of funding and the role of the funders for the present study and, if applicable, for the original study on which the present article is based	Page 19

*Give information separately for exposed and unexposed groups.

Note: An Explanation and Elaboration article discusses each checklist item and gives methodological background and published examples of transparent reporting. The STROBE checklist is best used in conjunction with this article (freely available on the Web sites of PLoS Medicine at http://www.plosmedicine.org/, Annals of Internal Medicine at http://www.annals.org/, and Epidemiology at http://www.epidem.com/). Information on the STROBE Initiative is available at www.strobe-statement.org.
